# MicroRNA-375 is a therapeutic target for castration-resistant prostate cancer through the PTPN4/STAT3 axis

**DOI:** 10.1038/s12276-022-00837-6

**Published:** 2022-08-30

**Authors:** Junqing Gan, Shan Liu, Yu Zhang, Liangzi He, Lu Bai, Ran Liao, Juan Zhao, Madi Guo, Wei Jiang, Jiade Li, Qi Li, Guannan Mu, Yangjiazi Wu, Xinling Wang, Xingli Zhang, Dan Zhou, Huimin Lv, Zhengfeng Wang, Yanqiao Zhang, Cheng Qian, MeiYan Feng, Hui Chen, Qingwei Meng, Xiaoyi Huang

**Affiliations:** 1 Department of Medical Oncology, Harbin Medical University Cancer Hospital, Harbin, Heilongjiang 150081 China; 2 Biotherapy Center, Harbin Medical University Cancer Hospital, Harbin, Heilongjiang 150081 China; 3 Department of Anesthesiology, Harbin Medical University Cancer Hospital, Harbin, Heilongjiang 150081 China; 4 Department of Neurosurgery, the First Affiliated Hospital of Zhengzhou University, Zhengzhou, Henan 450052 China; 5 Department of Gastrointestinal Medical Oncology, Harbin Medical University Cancer Hospital, Harbin, Heilongjiang 150081 China; 6 Department of Breast Surgery, Harbin Medical University Cancer Hospital, Harbin, Heilongjiang 150081 China; 7 Department of Pathology, Harbin Medical University Cancer Hospital, Harbin, Heilongjiang 150081 China; 8 Department of Urologic Surgery, Harbin Medical University Cancer Hospital, Harbin, Heilongjiang 150081 China; 9 NHC Key Laboratory of Cell Transplantation, Harbin Medical University, Harbin, Heilongjiang 150081 China

**Keywords:** miRNAs, Prostate cancer

## Abstract

The functional role of microRNA-375 (miR-375) in the development of prostate cancer (PCa) remains controversial. Previously, we found that plasma exosomal miR-375 is significantly elevated in castration-resistant PCa (CRPC) patients compared with castration-sensitive PCa patients. Here, we aimed to determine how miR-375 modulates CRPC progression and thereafter to evaluate the therapeutic potential of human umbilical cord mesenchymal stem cell (hucMSC)-derived exosomes loaded with miR-375 antisense oligonucleotides (e-375i). We used miRNA in situ hybridization technique to evaluate miR-375 expression in PCa tissues, gain- and loss-of-function experiments to determine miR-375 function, and bioinformatic methods, dual-luciferase reporter assay, qPCR, IHC and western blotting to determine and validate the target as well as the effects of miR-375 at the molecular level. Then, e-375i complexes were assessed for their antagonizing effects against miR-375. We found that the expression of miR-375 was elevated in PCa tissues and cancer exosomes, correlating with the Gleason score. Forced expression of miR-375 enhanced the expression of EMT markers and AR but suppressed apoptosis markers, leading to enhanced proliferation, migration, invasion, and enzalutamide resistance and decreased apoptosis of PCa cells. These effects could be reversed by miR-375 silencing. Mechanistically, miR-375 directly interfered with the expression of phosphatase nonreceptor type 4 (PTPN4), which in turn stabilized phosphorylated STAT3. Application of e-375i could inhibit miR-375, upregulate PTPN4 and downregulate p-STAT3, eventually repressing the growth of PCa. Collectively, we identified a novel miR-375 target, PTPN4, that functions upstream of STAT3, and targeting miR-375 may be an alternative therapeutic for PCa, especially for CRPC with high AR levels.

## Prostate cancer: stem cell treatment targets microRNA

A microRNA shown to promote prostate cancer growth can be targeted through a treatment derived from stem cells. Prostate cancer is lethal for many men, and its growth is promoted by testosterone. However, some “castration-resistant” strains keep growing even after treatments that reduce testosterone levels. Xiaoyi Huang at Harbin Medical University Cancer Hospital, China, and co-workers examined the role of microRNA-375 in the progression of castration-resistant prostate cancer. They found that microRNA-375 was over-expressed in cancer tissue samples and promoted tumor growth by interfering with a specific signaling pathway. The team applied a treatment comprising extracellular vesicles called exosomes that are derived from human stem cells and loaded with molecules that suppress microRNA-375. The treatment inhibited microRNA-375 and thereby repressed the cancer growth, while also reducing the cancer’s resistance to the testosterone-blocking drug enzalutamide.

## Introduction

Prostate cancer (PCa) accounts for one of the most common and lethal malignancies in men^1^. Currently, androgen deprivation therapy (ADT) is the first-line treatment for PCa. Unfortunately, most PCa cases gradually progress to metastatic castration-resistant prostate cancer (mCRPC) despite a good initial response^2^. When mCRPC occurs, an androgen receptor (AR) antagonist is usually prescribed^3^. However, due to aberrant activation of AR-dependent and AR-independent signaling, acquired resistance to AR antagonists has been proven to be another major challenge in PCa treatment^4^. Therefore, investigators still need to identify feasible therapeutic targets to hamper PCa progression_._

Gene therapy, either using short hairpin RNA or short interfering RNA to silence the expression of oncogenes, is emerging as an attractive treatment for various malignancies. MicroRNAs (miRNAs), which posttranscriptionally regulate gene expression levels pivotal for tumorigenesis, are feasible targets of gene therapy^5^. Our previous study demonstrated that the level of plasma exosomal miR-375 in CRPC patients was higher than that in castration-sensitive prostate cancer patients and predicted poor survival^6^. It has been reported that overexpression of miR-375 drives neuroendocrine differentiation states in PCa^7^. In CRPC, miR-375 can reduce cell sensitivity to docetaxel treatment by targeting SEC23A and YAP1^8^. This evidence suggests that miR-375 might play an onco-miR role in PCa, and antisense oligonucleotides (ASOs) targeting miR-375 may be a therapeutic option. However, conflicting findings have also been reported^9^, indicating that the mechanism of miR-375 in the development of PCa remains to be precisely elucidated and that the feasibility of targeting miR-375 as a curative option remains to be determined.

To enhance the in vivo transportation efficiency as well as the effects of therapeutic oligonucleotides, exosomes prepared from human umbilical cord mesenchymal stem cells (hucMSCs) are regarded as an ideal drug delivery vehicle^10^. Engineered exosomes loaded with 5-FU and miR-21i oligonucleotides were capable of inhibiting tumor growth while increasing apoptosis of 5-FU-resistant colorectal cancer cells^11^. Exosomes derived from hucMSCs loaded with miRNA-221 ASOs could dramatically inhibit the proliferation of colon cancer^12^. Traditionally, these therapeutic oligonucleotides are embedded in exosomes by electroporation or by genetic engineering of exosome-secreting cells. However, these procedures are time- and labor-consuming with low yield. Therefore, increasing the stability of therapeutic oligonucleotides and directly modifying exosomes seem to be promising solutions.

MiRNA ASOs synthesized with phosphorodiamidate morpholino oligonucleotides (PMOs) instead of traditional pentose phosphate nucleotides are uncharged oligonucleotides, render the oligomer exempt from nuclease digestion and inhibit miRNA activity by complementary binding to mature miRNA or pri-miRNA^13^, which is especially suitable for in vivo application in harsh digestive environments. Lobo et al. verified that PMOs against CD44v6-expressing tumors can increase gastric cancer (GC) sensitization to chemotherapy and delay GC progression^14^. Ma et al. also reported that PMO-mediated suppression of P53 sensitizes cancer cells to chemotherapy^15^.

In this study, we initially focused on the role of miR-375 in PCa development and enzalutamide resistance. After demonstrating that miR-375 contributes to PCa progression through phosphatase nonreceptor type 4 (PTPN4)/STAT3 signaling and could be a novel therapeutic target for PCa, we next loaded hucMSC-derived exosomes with miR-375 antisense PMOs (e-375i) by an in vitro engineering method and then evaluated the therapeutic and drug resensitizing effects of these miR-375 targeting complexes in vitro and in vivo.

## Materials and methods

### Preparation of cell lines, tissue specimens, and reagents

DU145, PC-3, LNCaP and BPH1 cells (the Institute of Biochemistry and Cell Biology of the Chinese Academy of Sciences, Shanghai, China) were maintained in RPMI 1640 (Biological Industries, Israel), and HEK-293 cells were maintained in DMEM (Biological Industries, Israel). Both basal media were supplemented with 10% FBS (Biological Industries, Israel) and 1% penicillin–streptomycin (Beyotime Biotechnology, Shanghai, China). All of the cells were incubated at 37 °C in a humidified environment supplied with 5% CO_2_.

We collected 30 PCa and 17 benign prostate hyperplasia tissues from Harbin Medical University Cancer Hospital.

Enzalutamide (Selleck, Houston, USA) was dissolved in DMSO (Sigma-Aldrich, USA) and stored at −20 °C until use. Sequences of PMOs (Gene Tools, https://www.gene-tools.com/, USA) were as follows: miR-375 antisense PMO (miR-375i): GCCTCACGCGAGCCGAACGAACAAA and negative control (NC): CCTCTTACCTCAGTTACAATTTATA. The oligomers were dissolved in PBS and stored at 4 °C until use.

### MiRNA in situ hybridization (ISH)

A tissue microarray composed of 150 specimens (3 healthy prostate, 54 paracancerous tissues and 93 prostate cancer tissues) (Shanghai Outdo Biotech, China) was used to assess miR-375 levels with a miRNAscope Kit RED (#324500, Advanced Cell Diagnostics, USA). Briefly, dewaxed tissue slide was hybridized with the customized miR-375-specific probe (Advanced Cell Diagnostics, USA) at 40 °C for 2 h. Then, the slide was subjected to signal amplification using an HD Reagent detection kit, and the hybridization signal was visualized with a DAB kit. A MiRNAscope positive control probe (#727871-S1, Advanced Cell Diagnostics, USA) and negative control probe (#727881-S1, Advanced Cell Diagnostics, USA) were used to ensure the interpretability of the hybridization. The signals were scored based on the following semi-quant guidelines: 0 (≤1 dot per cell), 1 (2–10 dots per cell and very few dot clusters), 2 (11–20 dots per cell and <25% dots were enclosed by clusters), and 3 (>20 dots per cell and >25% dots were enclosed by clusters).

### Bioinformatic analysis

EvmiRNA was used to assess miR-375 expression in different cancer-derived exosomes and microvesicles. The transcriptive data of miR-375 between disparate cancer tissues and normal control tissues were from The Cancer Genome Atlas (TCGA) and processed with R software (version 3.6.3). We also interrogated databases to calculate the unique expression levels of miR-375 (Starbase http://starbase.sysu.edu.cn/index.php) and PTPN4 (UALCAN http://ualcan.path.uab.edu/) in tumor and normal tissues) and the correlation between miR-375 and PTPN4. MiRWalk (http://mirwalk.umm.uni-heidelberg.de/), TargetScan (http://www.targetscan.org/vert_72/), StarBase, miRsystem (http://mirsystem.cgm.ntu.edu.tw/) and RNA22V2 (https://cm.jefferson.edu/rna22/Interactive/_)_were used to predict target genes of miR-375. The relationship between miR-375 and patients’ cancer stages of PCa was explored using Kruskal-Wallis R Test in the TCGA database (Normal: 52 cases, 6&7&8: 359 cases, 9&10:140 cases). The predictive power of miR-375 in PCa was expressed as the area under the receiver operator characteristic (ROC) curve using the pROC package. Gene set enrichment analysis (GSEA) was adopted to statistically explore differentially expressed genes relevant to the expression of PTPN4 in the TCGA database. First, the raw counts of differentially expressed PTPN4-related genes were downloaded from the TCGA-PRAD data portal by the Deseq2 package version 1.26.0. Then, genes with an adjusted *P* value <0.05 and log2-fold change >1.5 were considered statistically significant and were used for GSEA. The returned results were visualized via clusterProfiler package version 3.14.3 and ggplot2 package version 3.3.3. The enrichment pathways were evaluated by the *P* value and normalized enrichment score. GEPIA (http://gepia.cancer-pku.cn/) was performed to obtain the top 100 genes that had similar expression patterns as PTPN4 in prostate cancer. These similar genes were subjected to GO and KEGG analysis using R packages (clusterProfiler package 3.14.3 for enrichment analysis and org.Hs.eg.db package for ID Conversion).

### Real-time PCR

Total RNA from tissues or cultured cells was isolated using TRIzol reagent (Invitrogen Life Technologies, Carlsbad, USA). The miRNA and mRNA were reverse transcribed with a ReverTra Ace qPCR RT Kit (TOYOBO, Japan) in a reaction mixture containing a miR-specific stem-loop reverse transcription primer (miR-375 RT: GTCGTATCCAGTGCGTGTCGTGGAGTCGGCAATTGCACTGGATACGACTCACG) for miRNA or a universal poly (T) primer for mRNA. qRT-PCR was carried out using TransStartR Top Green qPCR SuperMix (TransGen Biotech, Beijing, China) and a SteponePlus Real-Time PCR system (Applied Biosystems, USA) to assess gene expression. GAPDH was used as a reference for mRNA levels, and U6 was used as a reference for miRNA levels. The sequences of the primers used are listed in Supplementary Table 1. Each PCR assay was repeated thrice.

### Western blot

Western blotting was performed as previously described^16^. All of the antibodies used in this article are listed in Supplementary Table 2.

### Dual-luciferase reporter gene assay

Fragments of the wild-type (WT) or mutant (MUT) 3′UTR of PTPN4 containing miR-375 binding sites were subcloned into the P-MIR-Report firefly luciferase vector. The sequence of pri-miR-375 was subcloned into the pCDH-CMV vector (pCDH-375). The cloning and mutation-introducing primers are listed in Supplementary Table 3. The WT or MUT firefly luciferase vector along with the Renilla luciferase vector (used as a protein loading control) were cotransfected with pCDH-375 or pCDH-empty (negative control) vector into HEK-293 cells. Forty-eight hours post-transfection, the cells were harvested and lysed. The luciferase activity of the lysate was detected with a Dual-Glo® Luciferase Assay System (Promega, Wisconsin, USA).

### Cell transfection

To upregulate or downregulate the expression of miR-375, DU145 and PC-3 cells were transfected with pSUPER-RETRO-Puro-miR-375, pSUPER-RETRO-Puro-NC, pHB-U6-MCS-PGK-PURO-miR-375 sponge (sp miR-375), and pHB-U6-MCS-PGK-PURO-NC (Hanbio Biotechnology, Shanghai, China) by using jetPRIME® (Polyplus-transfection® SA, Strasbourg, France). In brief, cells were seeded into six-well plates for 24 h before transfection. When the cell confluence reached 50–80%, the cells were transfected using jetPRIME®. After the medium was changed 4 h later, the cells were cultured for another 20 hours and screened with puromycin (DU145 1.5 µg/mL; PC-3 1 µg/mL). Colonies containing more than 50–100 cells were isolated, propagated, identified for the level of miR-375, and cultured for future use.

For the rescue experiment, cells overexpressing miR-375 were transfected with pcDNA3.1-PTPN4 recombinant or pcDNA3.1-empty plasmid (Invitrogen^TM^ Life Technologies, California, America). In parallel with PTPN4 overexpression, cells with miR-375 knockdown were transfected with siPTPN4 or siNC (Hanbio Biotechnology, Shanghai, China) by using jetPRIME®. The primers for subcloning and the target sequence of PTPN4 and the short interfering RNA targeting PTPN4 are presented in Supplementary Table 4.

### Cell proliferation assays

In the CCK-8 assays, transfected cells (5 × 10^3^ cells/well) were seeded. Cell proliferation was detected at 24, 48, 72, and 96 h using a CCK-8 kit (Dojindo, Kumamoto, Japan) following the manufacturer’s instructions. The absorbance at 450 nm was measured. Additionally, according to the manufacturer’s instructions, a Cell-Light TM EdU Apollo 567 In Vitro kit (Ribobio, Guangzhou, China) was utilized to assay EdU incorporation in the cells.

### Apoptosis analysis

Cells were harvested and washed three times, followed by treatment with 5 µL Annexin V-FITC (Dojindo, Japan) and 5 µL PI for 15 min in the dark at room temperature. Apoptotic cells were fractioned by flow cytometry (BD FACSAria^TM^ II, New Jersey, USA).

### Cell migration and invasion assays

Cells (5 × 10^5^/well) were maintained in six-well plates until they reached 100% confluence. A 10-µl pipette tip was applied to generate a wound, and the cell layer was then washed to remove detached cells. Next, the cells were incubated in serum-free RPMI 1640 at 37 °C for 24 h. The wound was photographed under a microscope at 0 h and 24 h, and the rate of closure was calculated with ImageJ software.

For the transwell assay, miR-375 -overexpressing or miR-375-silenced DU145 and PC-3 cells (5 × 10^4^) in serum-free RPMI 1640 were seeded into inner chambers with or without precoated Matrigel (Corning, New York, USA). RPMI 1640 containing 10% FBS was added to the outer chambers. After 16–24 h, the cells remaining in the inner chamber were removed with a cotton swab, and the cells migrated through the pores were fixed with 4% paraformaldehyde for 1 h. After the cells were stained with crystal violet for 1 hour, microscopic photographs were taken, and the number of migrated cells was counted.

### Enzalutamide sensitivity analysis

Cells (8 × 10^3^ cells/well) were incubated overnight. Then, they were exposed to different concentrations of enzalutamide (0, 1, 2, 4, 8, 16, 32, 64 µmol/L) and cultured for another 3 days. Cell viability was evaluated using a CCK-8 assay.

### Isolation and identification of hucMSCs

Fresh human umbilical cords were obtained from the Second Affiliated Hospital of Harbin Medical University with informed consent. Mesenchymal progenitor cells were cultured in serum-free MesenCult-ACF Plus Medium (05448, STEMCELL, Canada) immediately after the umbilical cord had been harvested. After perivascular Wharton’s jelly was incubated in 5% CO_2_ at 37 °C for 10–14 days until the cells (P0) were confluent around the colonization point, the hucMSCs were passaged, and the hucMSCs at the 4th passage were used for further analysis and experiments. Flow cytometry was used to detect the surface markers of the hucMSCs (refer to Supplementary Table 2 for the antibodies). To evaluate their potential for multiple differentiation, hucMSCs were cultured and induced in osteogenic, adipogenic, and chondrogenic differentiation medium (Biological Industries, Israel) for 6–21 days, followed by Alizarin red (MSC Osteo-Staining Kit, MC37C0-1.4, VivaCell Biosciences, China), Oil red O (MSC Adipo-Staining Kit, MC37A0-1.4, VivaCell Biosciences, China), and Alcian blue (MSC Chondro-Staining Kit, MC37B0-1.4, VivaCell Biosciences, China) staining.

### Exosome isolation and characterization

The serum-free supernatants of P4 hucMSCs were centrifuged at 4000 × *g* for 30 min, filtered through a 0.22 µm filter, and ultracentrifuged at 100,000 × *g* for 3 h at 4 °C (OptimaXPN-100 Ultracentrifuge). After the supernatant was removed, the pellet was suspended in PBS and ultracentrifuged for 2 h. The collected exosomes were resuspended in PBS and stored at −80 °C before use. Flow NanoAnalyzer N30 (NanoFCM Inc., Xiamen, China) was used to determine the size and concentration of exosomes. The morphological features of exosomes were detected with transmission electron microscopy (TEM; Hitachi 7500, Japan). Western blotting was used to assess surface markers, including CD63 (ab134045, Abcam), CD81 (ab79559, Abcam), and TSG101 (ab125011, Abcam).

### Cellular uptake of PKH67-labeled exosomes

To visualize the internalization of exosomes in DU145 and PC-3 cells, exosomes were stained with PKH67 (MINI67-1KT, Sigma-Aldrich, USA) in Diluent C. The mixture was filtered with a diffusiometer (Centrifugal Filter Units, Merck KGaA, Germany) to remove the excess dye. The PKH67-labeled exosomes were cocultured with DU145 and PC-3 cells for 24 h, followed by visualization under a confocal fluorescence microscope.

### Xenograft model

Four-week-old male BALB/c nude mice were obtained from Beijing Vital River Laboratory. A total of 4 × 10^6^ DU145 cells stably transfected with empty vector (Vector), miR-375 expression vector (miR-375) or miR-375 sponge vector (sp miR-375) were mixed with Matrigel (1:1) and subcutaneously injected into the backs of mice (8 mice per group). One week later, the mice were surgically castrated under anesthesia. When tumor volume were around ~50 mm^3^, each of the three groups of mice were again randomly stratified into two subgroups (4 mice per subgroup): (1) Vector (95% corn oil+5% DMSO, ip, 200 µl), (2) Vector + enzalutamide (10 mg/kg, ip, 200 µl), (3) miR-375 (95% corn oil + 5% DMSO, ip, 200 µl), (4) miR-375+ enzalutamide (10 mg/kg, ip, 200 µl), (5) sp miR-375 (95% corn oil+5% DMSO, ip, 200 µl), (6) sp miR 375+ enzalutamide (10 mg/kg, ip, 200 µl). Tumor size was measured twice a week, and the tumor volume was calculated with the formula: tumor volume = length × width^2^ × 0.5. The mice were sacrificed 30 days after subcutaneous injection. A fraction of the tumor tissue was fixed in 4% paraformaldehyde solution for hematoxylin and eosin (HE) and immunohistochemical staining analysis (IHC), and the rest was immediately stored at −80 °C for western blotting and real-time PCR.

For metastasis analysis, miR-375-overexpressing and miR-375-depleted DU145 cells (2 × 10^5^/100 µl) were injected into the mice through the tail vein (*n* = 4 per group). Eight weeks post injection, the mice were euthanized, and the lungs were removed. The metastatic tumor foci in the lungs were visualized and quantified by fixing the lungs in 4% paraformaldehyde, paraffin embedding the lungs and HE staining of serially sliced sections at 2 mm intervals.

To evaluate the in vivo function of e-375i, 4 × 10^6^/100 µl DU145 cells mixed with 100 µl Matrigel were subcutaneously injected into the backs of mice. Three days after injection, the mice were randomized into two groups (*n* = 5). Exosomes (1 × 10^9^) carrying NC oligonucleotides (5 nmol, e-NC group) or miR-375 antisense oligonucleotides (5 nmol, e-375i group) were intraperitoneally injected twice a week. Tumor volume and body weight were measured every 2 days until the mice were sacrificed 30 days after inoculation. The excised tumors were partially soaked in 4% paraformaldehyde and embedded in paraffin for IHC, and the rest were immediately stored at −80 °C for western blotting and real-time PCR.

### HE and IHC staining

The tissue sections were stained for H&E, and IHC for Ki-67, PTPN4, AR, E-cadherin and Vimentin was performed as previously described^17^. Image-Pro Plus 6.0 was utilized to assess the average integrated optical density of the staining when the IHC images were loaded into the software.

### Statistical analysis

Data analysis was performed using GraphPad Prism 8. The data are presented as the mean ± SEM. Student’s *t* test or one-way analysis of variance was utilized to calculate statistical significance. The association between miR-375 and Gleason score was statistically determined with the Kruskal-Wallis test and Dunn’s test. Statistical results with *P < 0.05; **P < 0.01; ***P < 0.001; ****P < 0.0001 were statistically significant.

## Results

### MiR-375 expression was aberrantly increased in PCa tissues

**Fig. 1:**
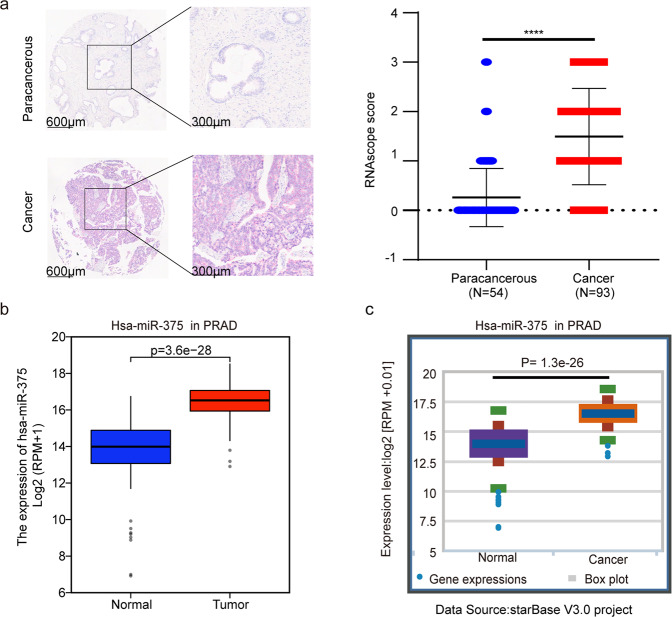
MiR-375 level in PCa. **a** Representative images of RNAscope technology for miR-375 in paracancerous and cancer tissues. The red dots indicate the target molecules (left). Statistical analysis of the RNAscope scores (right). MiR-375 expression in PCa from the TCGA (**b**) and the StarBase database (**c**). PRAD prostate cancer.

After determining the high level of plasma exosomal miR-375 in CRPC patients^6^, we investigated the possible origin of the circulating miR-375. RNAscope was carried out by using a miR-375-specific ISH probe in a PCa tissue chip, which consists of 54 paracancerous and 93 cancer tissues. The representative ISH image is shown in Fig. 1a on the left. According to our grading criteria, the mean miR-375 level in PCa tissues was up to sixfold higher than that in paracancerous tissues (1.50 ± 0.97 versus 0.26 ± 0.58, *P* < 0.0001). Fig. 1a right). MiR-375 was also highly expressed in PCa-derived exosomes, as evaluated by EvmiRNA (Supplementary Fig. 1a). In line with our RNAscope results, comparison using TCGA data and those in StarBase revealed that miR-375 was prominently overexpressed in PCa tissues (Fig. 1b and c). The relationship between miR-375 and PCa patients’ cancer stages was evaluated in the TCGA database, revealing that miR-375 was positively associated with the Gleason score (Supplementary Fig. 1b) and possessed high diagnostic power for PCa with an area under the curve of 0.963 (95% CI, 0.936–0.990, Supplementary Fig. 1c). The above evidence indicated that the elevated plasma exosomal miR-375 in PCa patients was likely from cancerous tissue. We next aimed to clarify the roles of miR-375 in castration-resistant prostate cancer progression and the extent to which miR-375 can serve as a therapeutic target; therefore, we chose two androgen-independent PCa cell lines, DU145, and PC-3, in which miR-375 was mildly expressed^9^, for subsequent experiments.

### MiR-375 promoted PCa development

**Fig. 2:**
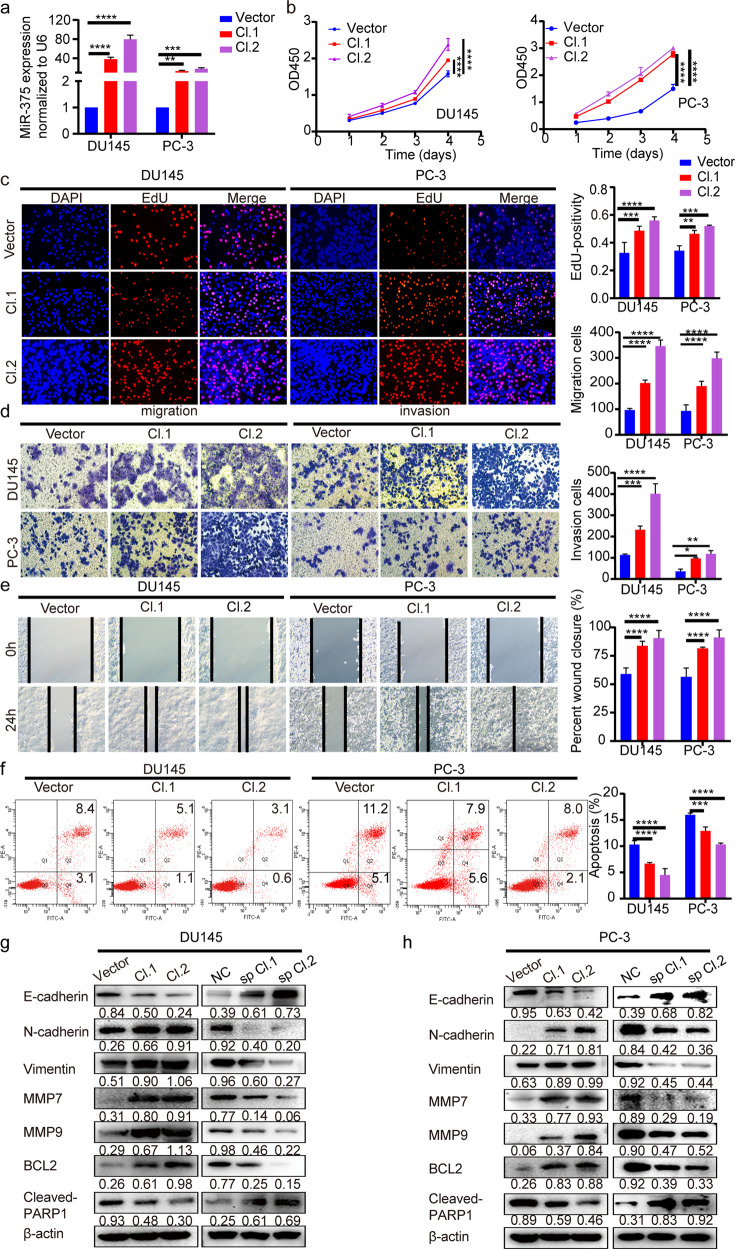
MiR-375 promoted PCa cell proliferation, migration, and invasion but restrained apoptosis. **a** MiR-375 overexpression in DU145 and PC-3 cells was verified via qRT-PCR after these cells were stably transfected with pSUPER-RETRO-Puro-miR-375 recombinant vector or pSUPER-RETRO-Puro-empty vector (serving as a control). CCK-8 and EdU incorporation assays were performed to assess the cell proliferation (**b**) and DNA synthesis rate (**c**) in miR-375-overexpressing DU145 and PC-3 cells. Transwell (**d**) and wound healing assays (**e**) were performed to confirm the effects of miR-375 overexpression on cell migration and invasion. **f** Flow cytometric analysis of the apoptotic rate in DU145 and PC-3 cells with or without miR-375 overexpression. **g**, **h** The protein levels of EMT and apoptosis markers in miR-375-overexpressing or miR-375-knockdown DU145 and PC-3 cells compared with the respective control cells. Cl. 1: Cell colony 1 transfected with pSUPER-RETRO-Puro-miR-375 recombinant vector. Cl. 2: Cell colony 2 transfected with pSUPER-RETRO-Puro-miR-375 recombinant vector. Vector: cell colony transfected with pSUPER-RETRO-Puro-empty vector (served as control). sp Cl.1: cell colony 1 transfected with pHB-U6-MCS-PGK-PURO-miR-375 sponge vector. Sp Cl.2: cell colony 2 transfected with pHB-U6-MCS-PGK-PURO-miR-375 sponge vector. NC: cell colony transfected with pHB-U6-MCS-PGK-PURO-empty vector (served as control).

To investigate the role of miR-375 in the malignant characteristics of PCa cells, we overexpressed or knocked down miR-375 in DU145 and PC-3 cells, respectively (Fig. 2a, Supplementary Fig. 2a). CCK-8 and EdU assays were performed to determine cell proliferation. As shown in Fig. 2b, c, upregulation of miR-375 significantly promoted DU145 and PC-3 cell growth. Transwell and wound healing assays revealed that miR-375 overexpression facilitated migration and invasion (Fig. 2d, e) while inhibiting apoptosis of PCa cells (Fig. 2f). These effects could be completely reversed when the miR-375 sponge was introduced into DU145 and PC-3 cell lines (Supplementary Fig. 2b–f).

At the molecular level, miR-375 overexpression promoted the expression of N-cadherin, Vimentin, MMP7, MMP9, and BCL2 suppressed E-cadherin and cleaved-PARP1 (Fig. 2g left, 2h left). This expression signature could be substantially reversed when DU145 and PC-3 cells were transfected with a miR-375 sponge vector (Fig. 2g right, 2h right). The above results collectively suggested that miR-375 could modulate the expression of EMT and apoptosis markers, which in turn promoted PCa development.

### MiR-375 targeted PTPN4 directly

**Fig. 3:**
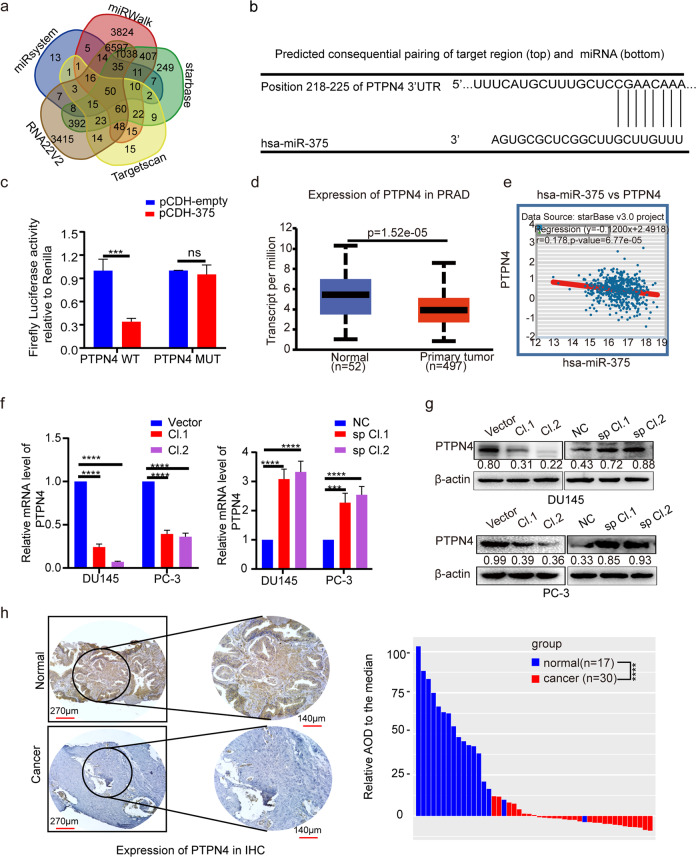
MiR-375 targeted and negatively regulated PTPN4 in PCa. **a** Potential target genes were predicted using the miRsystem, miRWalk, StarBase, TargetScan and RNA22v2 databases. A total of 50 genes were found to be shared in the five databases. **b** The potential binding site of miR-375 with the 3′-UTR of PTPN4 was predicted using the TargetScan database. Complementary sequences consisting of 8 nt were aligned and presented. **c** A luciferase assay was conducted to validate the direct binding and modulating function of miR-375 to the 3′-UTR of PTPN4. PCDH-empty: HEK-293 cells transfected with the pCDH-CMV vector. PCDH-375: HEK-293 cells transfected with pCDH-CMV vector that was subcloned with a fragment of pri-miR-375. PTPN4 WT: HEK-293 cells cotransfected with the P-MIR-Report firefly luciferase vector inserted with a fragment of the wild-type 3’-UTR of PTPN4 mRNA. PTPN4 MUT: HEK-293 cells cotransfected with the P-MIR-Report firefly luciferase vector subcloned with the 3′-UTR of PTPN4 mRNA mutated at the complementary site. **d** The expression of PTPN4 in PCa tissues compared with normal prostate tissues from the UALCAN database. **e** The correlation between miR-375 level and PTPN4 in PCa, statistically processed by StarBase. *r* = −0.178, *p* = 6.77e-5. PTPN4 at the mRNA level (**f**) and protein level (**g**), as determined by qRT-PCR and western blot in DU145 and PC-3 cells after miR-375 was overexpressed or knocked down. **h** IHC illustrating the differential expression of PTPN4 between 30 PCa and 17 normal tissues. The results show a representative IHC image (left) and quantitative analysis calculated by Image-Pro Plus version 6.0 software based on the 47 IHCs (right). AOD: average integrated optical density.

To dissect the underlying mechanisms of miR-375 in promoting PCa progression, we first determined the direct target of miR-375. By using five different target gene prediction databases, we identified 50 intersecting genes (Fig. 3a, Supplementary Table 5) falling into three subgroups according to the cumulative weighted context++ score ranging from 0 to −0.1, between −0.1 and −0.2, and less than −0.2. We randomly chose one gene from each subgroup (PTPN4, EIF4G3, and UBE3A) for downstream validation. The miR-375 binding site in the 3′UTR of PTPN4, EIF4G3 and UBE3A mRNA, predicted by TargetScan, is presented in Fig. 3b and Supplementary Fig. 3a, b. As shown in Fig. 3c and Supplementary Fig. 3c, d, miR-375 only decreased the luciferase activity in HEK-293 cells cotransfected with reporter vectors harboring wild type, but not the mutant, 3′-UTR of PTPN4, EIF4G3 and UBE3A mRNA (*P* < 0.001, *P* < 0.01 or *P* < 0.001, respectively), suggesting miR-375 directly targeted and inhibited these three genes. Of note, PTPN4 was suppressed in prostate cancer tissues in the UALCAN database (Fig. 3d), which was negatively correlated with miR-375 expression, as illustrated in StarBase (Fig. 3e). Other than PTPN4, no statistical significance was found regarding EIF4G3 or UBE3A expression when prostate cancer tissues were compared to normal tissues in the UALCAN database (Supplementary Fig. 3e). Moreover, no statistical significance was found between the expression of EIF4G3 or UBE3A and miR-375, as evaluated in StarBase (Supplementary Fig. 3f). Regarding PC-3 and DU145 cells, qRT-PCR results demonstrated that only PTPN4 mRNA was significantly and reversely altered as miR-375 was overexpressed and knocked down (Fig. 3f and Supplementary Fig. 3g, h). Likewise, protein levels of PTPN4 were reduced post miR-375 overexpression but increased upon miR-375 downregulation (Fig. 3g). Therefore, we chose PTPN4, a pivotal tumor inhibitor^18^, for subsequent experiments. In the Human Protein Atlas, significantly depressed PTPN4 was also found in PCa tissues compared with normal tissues (Supplementary Fig. 3i). To further verify the bioinformatic findings, we performed IHC in 17 benign prostate hyperplasia and 30 PCa tissues. Indeed, PTPN4 was mainly located in the cytoplasm and had significantly higher expression in benign tissues than in PCa tissues (65.43 ± 28.07 versus 13.46 ± 5.39, *p* < 0.0001; Fig. 3h).

Genes with similar expression patterns are generally acknowledged to be functionally related^19^. We next identified 100 PTPN4-related genes (Supplementary Table 6) in GEPIA and annotated their cellular functions via GO analysis. We found that PTPN4, along with its similar genes, was putatively related to the production of miRNAs, involved in gene silencing by miRNAs and the negative regulation of the cell cycle process (Supplementary Fig. 3j–l). In KEGG analysis, PTPN4 was significantly related to prostate cancer and miRNAs in cancer (Supplementary Fig. 3m). Together with the finding that PTPN4 acts as a tumor inhibitor, it is suggested that miR-375 may play an important role in miRNA-mediated PCa progression by negatively regulating PTPN4.

### MiR-375 regulated PCa progression via the PTPN4-STAT3 pathway

**Fig. 4:**
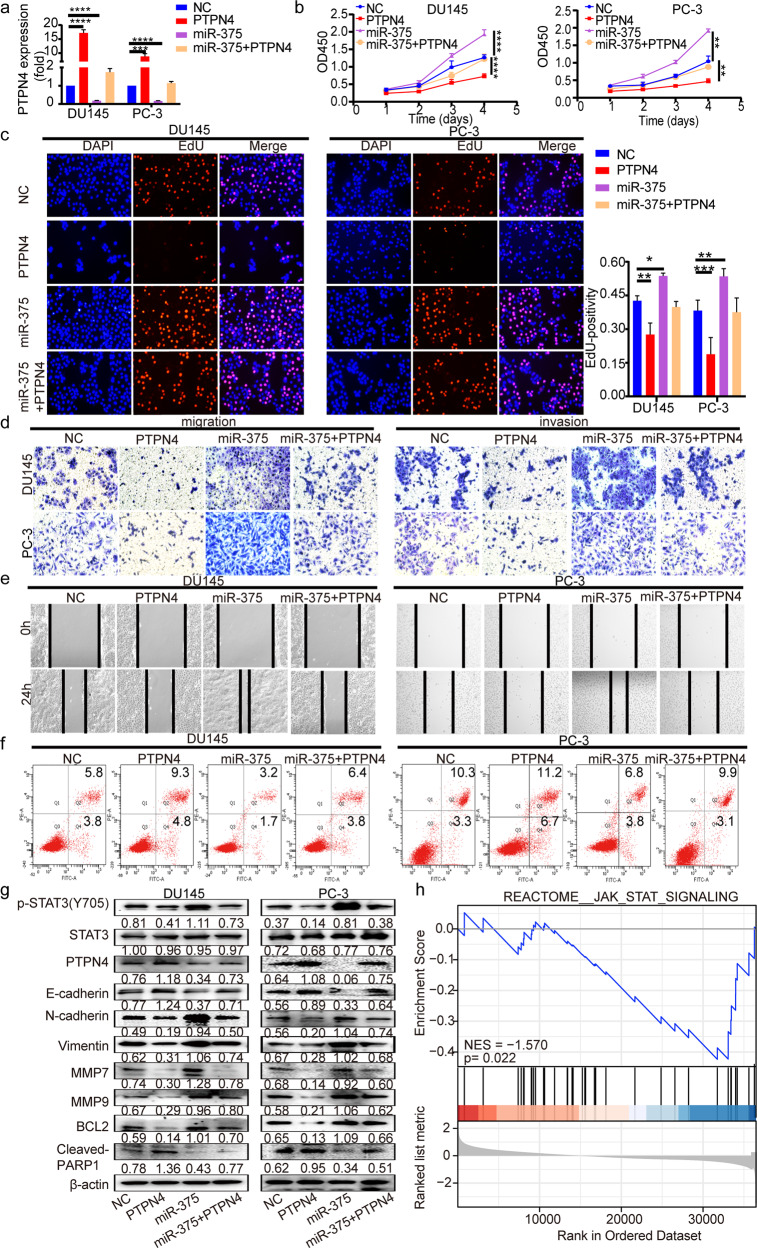
MiR-375 overexpression promotes proliferation, invasion, and migration and inhibits apoptosis via the PTPN4-STAT3 pathway. **a** qRT-PCR was performed to measure the mRNA expression of PTPN4 post PTPN4 and/or miR-375 overexpression. **b** CCK-8 and **c** EdU incorporation assays were conducted to test the proliferation of DU145 and PC-3 cells in response to overexpression of miR-375 and/or PTPN4. **d** Transwell and **e** wound-healing assays were performed to determine the migration and invasion of DU145 and PC-3 cells in response to overexpression of miR-375 and/or PTPN4. **f** Apoptosis analysis of DU145 and PC-3 cells in response to miR-375 and/or PTPN4 overexpression. **g** Western blotting was conducted to evaluate p-STAT3, PTPN4, EMT- and apoptosis-related markers of DU145 and PC-3 cells in response to overexpression of miR-375 and/or PTPN4. **h** GSEA using the data deposited in the TCGA database suggested that PTPN4 negatively regulated the JAK-STAT pathway. NC: cells stably transfected with pSUPER-RETRO-Puro-empty vector and transiently transfected with pcDNA3.1-empty vector. PTPN4: cells stably transfected with pSUPER-RETRO-Puro-empty vector and transiently transfected with pcDNA3.1-PTPN4 recombinant vector. MiR-375: cells stably transfected with pSUPER-RETRO-Puro-miR-375 recombinant vector and transiently transfected with pcDNA3.1-empty vector. MiR-375 + PTPN4: cells stably transfected with pSUPER-RETRO-Puro-miR-375 recombinant vector and transiently transfected with pcDNA3.1-PTPN4 vector.

To confirm that PTPN4 is a mediator accounting for miR-375-elicited tumor development, rescue assays were carried out. Figure 4a, g shows that both the mRNA and protein levels of PTPN4, which had been suppressed by induction of miR-375, were recovered upon transfection of the PTPN4 expression vector in miR-375-overexpressing DU145 and PC-3 cells. The promoting effects on cell proliferation, migration, and invasion and suppressing effect on apoptosis upon miR-375 overexpression were significantly attenuated by overexpression of PTPN4 (Fig. 4b–f and Supplementary Fig. 4a–d). Overexpression of PTPN4 also rescued the expressional alteration of the apoptosis markers (cleaved-PARP1 and BCL2) and EMT markers (E-cadherin, N-cadherin, Vimentin, MMP7, and MMP9) brought upon by enhanced miR-375 expression (Fig. 4g). In the case of potential artifacts, we knocked down PTPN4 expression in PCa cells whose miR-375 expression was depleted with a sponge vector. After screening for the eligible siRNA molecule in the two cell lines, we selected siPTPN4#1 for the following experiment because it displayed the best inhibition effect as shown in Supplementary Fig. 5a. Furthermore, the results in Supplementary Fig. 5b and h show that both the mRNA and protein levels of PTPN4, which had been upregulated by inhibition of miR-375, were significantly inhibited upon transfection of siPTPN4#1 in miR-375-suppressed DU145 and PC-3 cells. As shown in Supplementary Fig. 5c–g and Supplementary Fig. 6a–d, the inhibitory effects of miR-375 depletion on the progression of PCa were also rescued by PTPN4 silencing.

STAT3 signaling is a pivotal downstream pathway that responds to PTPN4 phosphorylation^20^. Our GSEA results also revealed that PTPN4 function in PCa might be related to inactivation of the JAK-STAT signaling pathway (Fig. 4h). Therefore, we proposed that the STAT3 pathway was involved in miR-375-modulated PCa progression. Indeed, overexpression of PTPN4 substantially reversed the enhancing effect of miR-375 on p-STAT3 expression (Fig. 4g), while suppression of PTPN4 reversed the inhibited level of p-STAT3 that responded to miR-375 depletion (Supplementary Fig. 5h). These findings indicated that PTPN4 antagonized the function of miR-375, which was overexpressed in prostate cancer, by inhibiting the STAT3 pathway.

### MiR-375 promoted PCa development in vivo

**Fig. 5:**
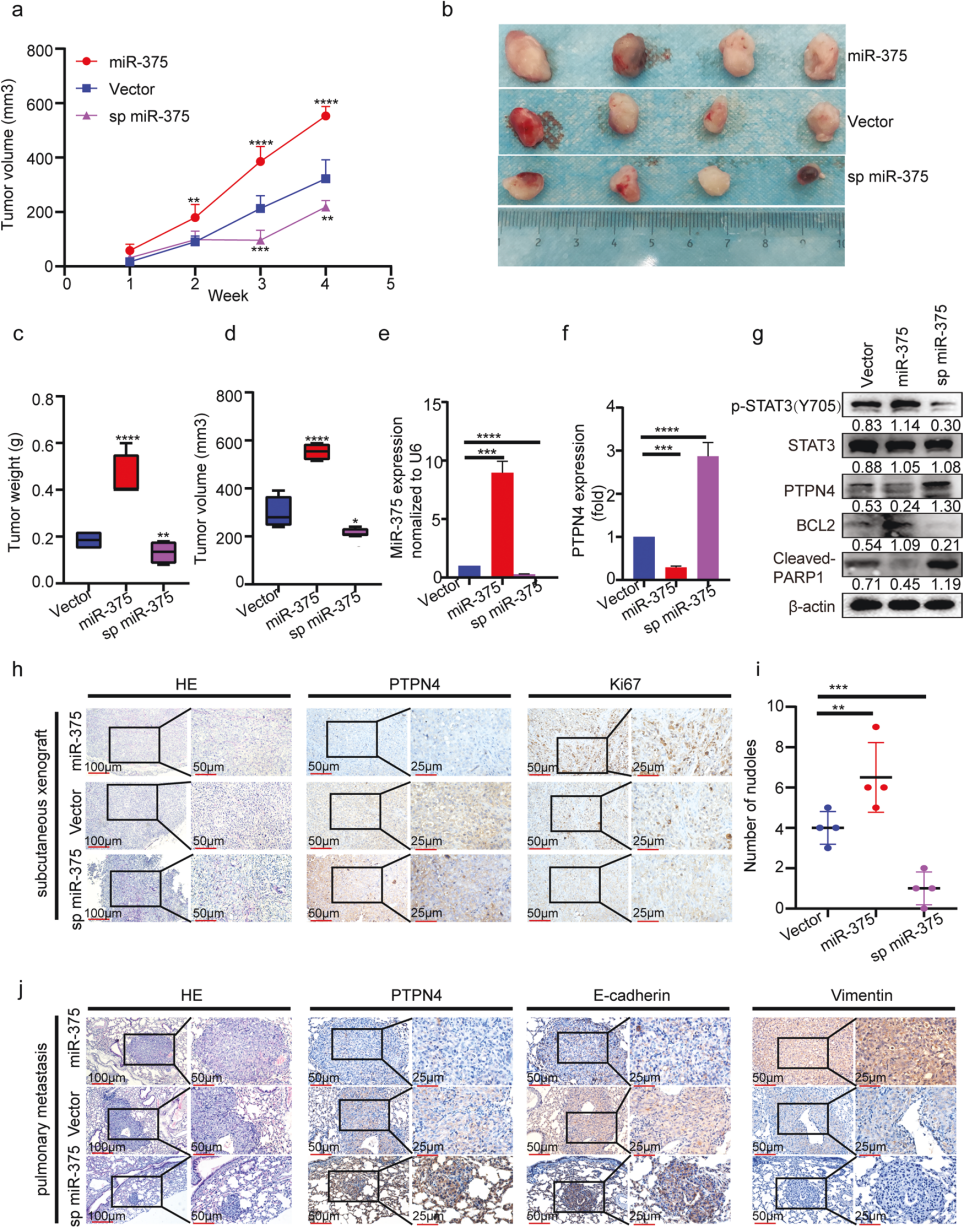
MiR-375 overexpression promoted tumor growth and metastasis in vivo. **a** Tumor volume during follow-up for 4 weeks. **p < 0.01, ***p < 0.001, ****p < 0.0001 vs. vector group. **b** Representative images of tumors in nude mice at the end timepoint. **c** Final tumor weight, **d** final tumor volume, **e** the expression of miR-375, and **f** mRNA level of PTPN4 were determined by qRT-PCR in tumor tissues at the endpoint of follow-up. **g** Western blotting was performed to determine the expression of p-STAT3, PTPN4, BCL2 and cleaved-PARP, and **h** IHC analysis of the expression of PTPN4 and Ki67 in DU145 tumor tissues as the expression of miR-375 was manipulated. **i** Quantification of the metastatic nodules calculated from the discontinuous lung sections. **j** HE staining and IHC analysis of the expression of PTPN4, E-cadherin and Vimentin using metastatic lung tissue. Vector: DU145 cells stably transfected with pSUPER-RETRO-Puro-empty vector and pHB-U6-MCS-PGK-PURO-empty vector. MiR-375: DU145 cells stably transfected with pSUPER-RETRO-Puro-miR-375 vector and pHB-U6-MCS-PGK-PURO-empty vector. Sp miR-375: DU145 cells stably transfected with pHB-U6-MCS-PGK-PURO-miR-375 sponge vector and pSUPER-RETRO-Puro-empty vector.

To confirm our in vitro findings, DU145 cells with miR-375 overexpression or depletion were used to establish a xenograft nude mouse model (Supplementary Fig. 6e). As illustrated in Fig. 5a–d, upregulation of miR-375 notably facilitated PCa growth, whereas miR-375 depletion demonstrated the opposite effect in vivo. In line with our in vitro findings, our in vivo study confirmed that miR-375 expression was strikingly enhanced in miR-375-overexpressing tumors and suppressed in miR-375-knockdown tumors (Fig. 5e), consistent with PTPN4 exhibiting the opposite trend at both the mRNA and protein levels (Fig. 5f, g). Additionally, the expression of p-STAT3 and BCL2 was significantly increased, while cleaved PARP was decreased in response to miR-375 upregulation (Fig. 5g). The corroborating IHC analysis results showed that Ki67 expression was prominently enhanced in the miR-375-overexpressing group compared to the control group (Fig. 5h).

In our pulmonary metastasis model, we did not find statistical significance regarding the metastatic nodules at the surface of the lung among all groups, and only mild to moderate collapses were found in the vector control group or miR-375 group (Supplementary Fig. 6f). However, scrutinizing the discontinuous lung sections and HE staining, we found obvious increases both in numbers and in the area of the metastatic foci in the miR-375-overexpressing tumor compared to the vector control tumor (Fig. 5i, j). Furthermore, in Fig. 5j, the IHC assay showed that PTPN4 and E-cadherin expression were markedly decreased, while Vimentin expression was increased upon miR-375 overexpression. All of the above effects elicited by miR-375 overexpression were substantially reversed when miR-375 was depleted in the miR-375 sponge tumors, which confirmed our in vitro studies on metastasis and the direct modulating role of miR-375 on PTPN4.

### HucMSC-derived exosomes carrying miR-375 antisense PMO (e-375i) inhibited PCa progression in vitro and in vivo

**Fig. 6:**
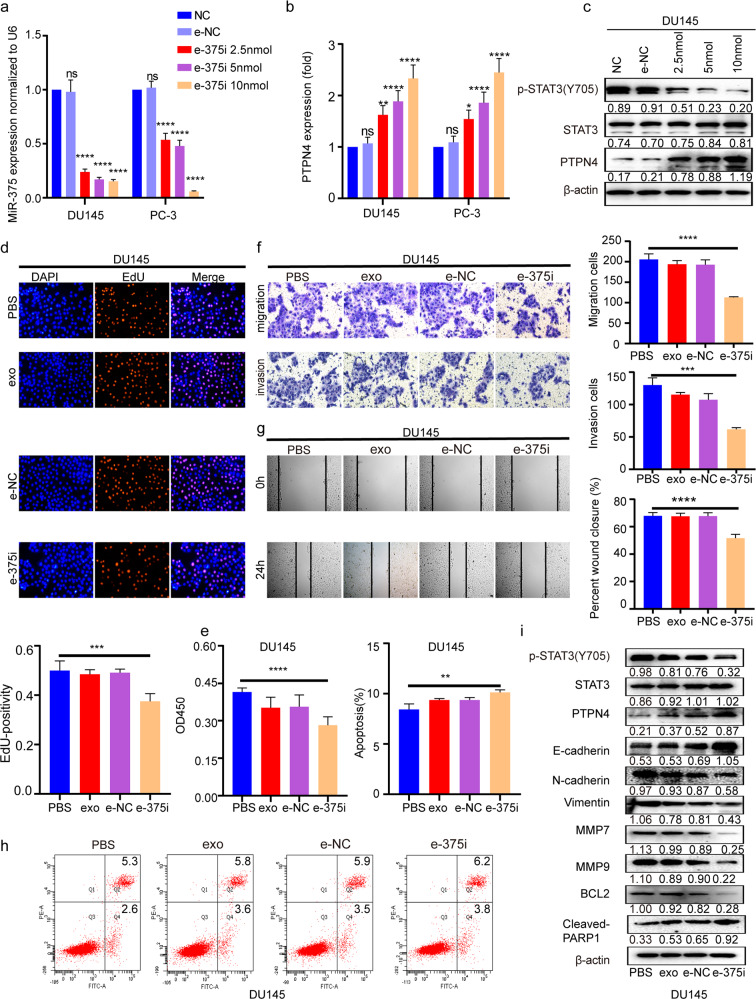
E-375i inhibited PCa proliferation, migration, and invasion while promoting apoptosis in DU145 cells. qRT-PCR illustrating miR-375 AMO loaded and intercellular transferred by hucMSC derived exosomes inhibited expression of miR-375 in DU145 and PC-3 cells (**a**), diminished the inhibitory effect on PTPN4 (**b**) in a dose-dependent manner determined by qRT-PCR, as well as downregulated the protein expression of p-STAT3 and upregulated PTPN4 (**c**) in a dose-dependent manner in DU145 cells, which were determined by western blot. **d** EdU incorporation and **e** CCK-8 assays were used to assess the role of miR-375 AMO-loaded exosomes on the proliferation of DU145 cells. **f** Transwell and **g** wound-healing assays were applied to test the effects of miR-375 AMO on cell migration and invasion in DU145 cells. **h** Flow cytometry was conducted to test the function of miR-375 AMO on the apoptotic rate of DU145 cells. **i** The effect of miR-375 AMO on the protein expression of p-STAT3, EMT and apoptosis markers in DU145 cells. PBS: cells treated with PBS that served as suspension control of exosomes. NC: scramble oligonucleotide. exo: exosomes suspended in PBS. e-NC: exosomes loaded with scramble oligonucleotides. e-375i: exosomes loaded with miR-375 AMO.

To test whether exosomes can efficiently transport miR-375 antisense PMO into target cells and counter miR-375 function, hucMSCs were grown in serum-free medium and characterized by tri-lineage differentiation assays and the signature of surface markers at the 4th passage (Supplementary Fig. 7a, b). Exosomes with diameters of 30–150 nm, as determined by TEM, were isolated from the supernatant of the cell culture (Supplementary Fig. 7c). After their size distribution and concentration were determined by NanoFlow (Supplementary Fig. 7d), exosome-specific markers were further verified with western blotting (Supplementary Fig. 7e). The exosomes were coincubated with miR-375 antisense PMO to form the e-375i complex at room temperature. Next, we determined whether the miR-375i PMO could be transferred into PCa cells by exosomes. As shown in Supplementary Fig. 7f, PKH67-labeled exosomes (green) could be efficiently internalized by PCa cells. Treatment with e-375i significantly inhibited miR-375 levels and p-STAT3 but upregulated PTPN4 in a dose-dependent manner (Fig. 6a–c). E-375i also significantly reduced PCa cell proliferation, migration, and invasion while promoting apoptosis (Fig. 6d–h). Moreover, e-375i treatment restrained the expression of N-cadherin, Vimentin, MMP7, MMP9 and BLC2 but upregulated the expression of PTPN4, E-cadherin and cleaved-PARP1 (Fig. 6i). The effects of e-375i derived from DU145 cells could also be duplicated in PC-3 cells (Supplementary Fig. 8a–f). Therefore, our results demonstrated that miR-375 antisense PMOs could be transported into PCa cells by exosomes and that e-375i significantly inhibited PCa development in vitro.

**Fig. 7:**
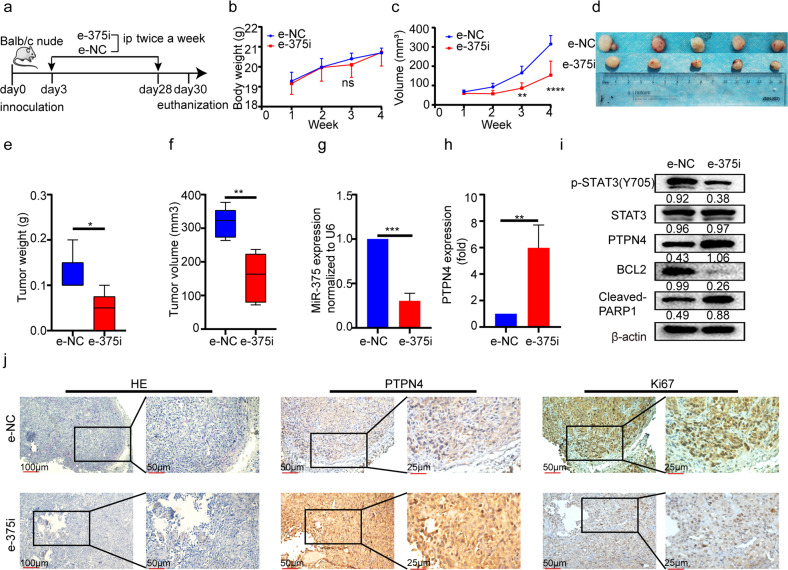
E-375i inhibited PCa proliferation in vivo. **a** The flow chart showing the study design. **b** The bodyweight of the animals and **c** the tumor volume during the follow-up. **d** Representative images of tumors, **e** tumor weight, and **f** tumor volume at the endpoint of observation. **g** The expression of miR-375 and **h** mRNA level of PTPN4 were tested by qRT-PCR in endpoint tumor tissues. **i** Western blot showing the expression of p-STAT3, PTPN4, BCL2 and cleaved-PARP1 in tumor tissues. **j** IHC analysis of the expression of PTPN4 and Ki67 in the two groups.

To test the in vivo effects of e-375i, a xenograft model was established (Fig. 7a). During the follow-up, the body weight of the animals remained stable as the tumors were regularly treated with e-375i (Fig. 7b). The tumor size and weight were markedly decreased (Fig. 7c–f), suggesting that e-375i was safe and effective in vivo for therapeutic purposes. Moreover, in the e-375i-treated tumors, miR-375 level depletion was sustained (Fig. 7g), while the PTPN4 level was markedly increased at both the protein and mRNA levels (Fig. 7h–j). Consistent with the results in Fig. 6i, e-375i treatment resulted in reduced expression of p-STAT3 and BCL2, increased cleaved-PARP1, and significantly decreased Ki67 in tumor tissue (Fig. 7i, j). Overall, e-375i was capable of regulating the PTPN4/STAT3 pathway and continuously suppressing PCa cell proliferation both in vitro and in vivo.

### MiR-375 regulated enzalutamide resistance via the PTPN4-STAT3 pathway

**Fig. 8:**
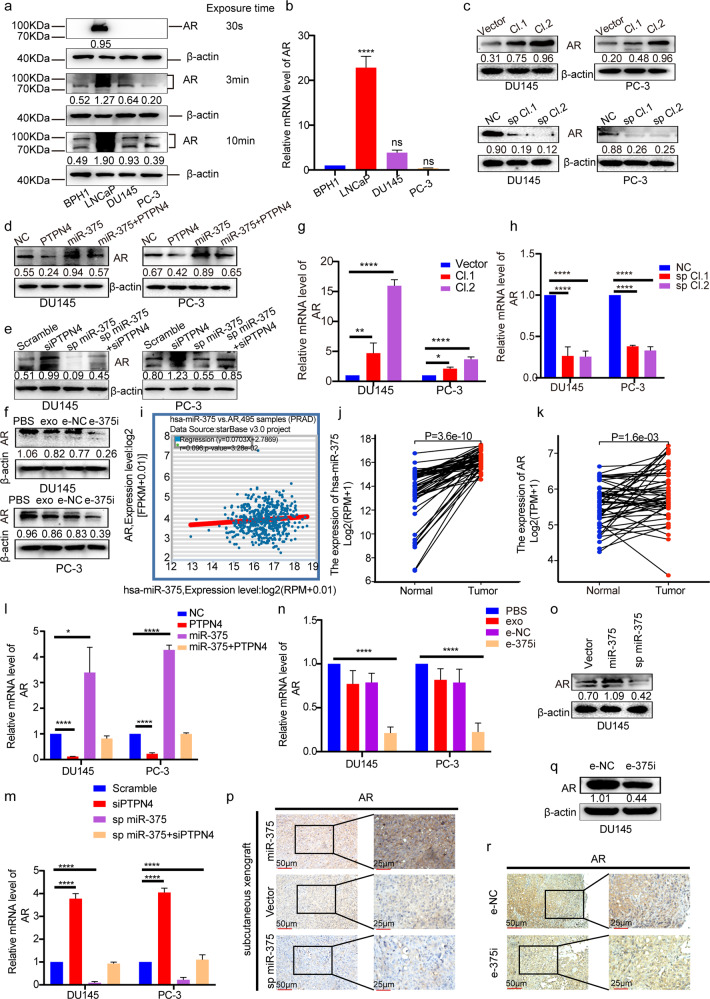
MiR-375 regulated the expression of AR by targeting the PTPN4-STAT3 signaling pathway. AR protein (**a**) and mRNA (**b**) levels in BPH and PCa cell lines. **c** The protein levels of AR in miR-375-overexpressing or miR-375-knockdown DU145 and PC-3 cells compared with the control cells. Western blotting was conducted to evaluate AR expression in DU145 and PC-3 cells in response to **d** overexpression of miR-375 and/or PTPN4 and **e** suppression of miR-375 and/or PTPN4. **f** The effect of miR-375 AMO on the protein expression of AR in DU145 and PC-3 cells. The mRNA expression of AR in DU145 and PC-3 cells was verified via qRT-PCR after miR-375 was overexpressed (**g**) or knocked down (**h**). **i** The correlation between miR-375 levels and AR in PCa, statistically processed by StarBase. r = 0.096, *p* = 3.28e-2. **j** The expression of miR-375 and **k** AR in PCa tissues compared with paired normal prostate tissues from TCGA database. The mRNA levels of AR in PTPN4- and/or miR-375-overexpressing (**l**) and PTPN4- and/or miR-375-suppressed (**m**) DU145 and PC-3 cells, determined by qRT-PCR. **n** qRT-PCR results showing the transcription levels of AR in DU145 and PC-3 cells after treatment with e-375i or the controls. **o** Western blot showing the expression of AR in DU145 tumor tissues as the expression of miR-375 was manipulated. **p** IHC analysis of AR expression in DU145 tumor tissues as miR-375 expression was manipulated. **q** Western blotting showing the expression of AR in tumor tissues treated with e-375i. **r** IHC analysis of the expression of AR in the e-375i and e-NC groups. Scramble: cells stably transfected with pHB-U6-MCS-PGK-PURO-empty vector and transiently transfected with siNC. SiPTPN4: cells stably transfected with pHB-U6-MCS-PGK-PURO-empty vector and transiently transfected with siPTPN4 vector. Sp miR-375: cells stably transfected with pHB-U6-MCS-PGK-PURO-miR-375 sponge and transiently transfected with siNC. Sp miR-375 + siPTPN4: cells stably transfected with pHB-U6-MCS-PGK-PURO-miR-375 recombinant vector and transiently transfected with siPTPN4.

It has been reported that androgen receptor (AR), the key player in PCa resistance to the second-generation AR antagonist enzalutamide^21^, is a downstream effector of STAT3 signaling^22^. Although the mainstream view is that DU145 and PC-3 cells are devoid of AR expression^23^, a study solidified the positive expression of AR in the two cell lines^24^. Likewise, as illustrated in Fig. 8a, b, we also observed mild expression of AR protein and mRNA in DU145 and PC-3 cells, whose levels could be increased upon miR-375 overexpression but decreased upon miR-375 inhibition (Fig. 8c). Additionally, in these cell lines, PTPN4 overexpression reversed miR-375-induced AR upregulation (Fig. 8d); PTPN4 inhibition reversed miR-375 suppression-induced AR downregulation (Fig. 8e). Notably, in vitro application of e-375i also restrained AR expression (Fig. 8f).

We next investigated the factors that contributed to AR expression changes after miR-375 introduction. First, we checked whether miR-375 modulated AR expression at the transcriptional level by qRT-PCR. We found that when miR-375 was overexpressed, the level of AR mRNA was significantly increased and vice versa (Fig. 8g, h). In accordance with our observations, data from StarBase revealed that miR-375 was positively correlated with AR (Fig. 8i). Based on the data derived from 52 pairwise PCa specimens in the TCGA database, both miR-375 and AR mRNA were remarkably upregulated in PCa cancerous tissues compared with matched normal tissues (Fig. 8j, k). Next, we found that forced expression of PTPN4 was able to substantially reverse the enhancing effect of miR-375 on AR mRNA (Fig. 8l), while inhibition of PTPN4 was able to reverse the suppression effect of miR-375 depletion on AR mRNA (Fig. 8m). As expected, e-375i treatment attenuated the expression of AR mRNA (Fig. 8n). Our in vitro findings confirmed that AR was significantly enhanced in miR-375-overexpressing tumors but decreased in miR-375-knockdown tumors (Fig. 8o). The western blot-based readouts were further corroborated by the IHC assay results (Fig. 8p). In the e-375i-treated PCa tumors, significantly reduced AR expression (Fig. 8q, r) was observed, consistent with the in vitro results shown in Fig. 8f.

**Fig. 9:**
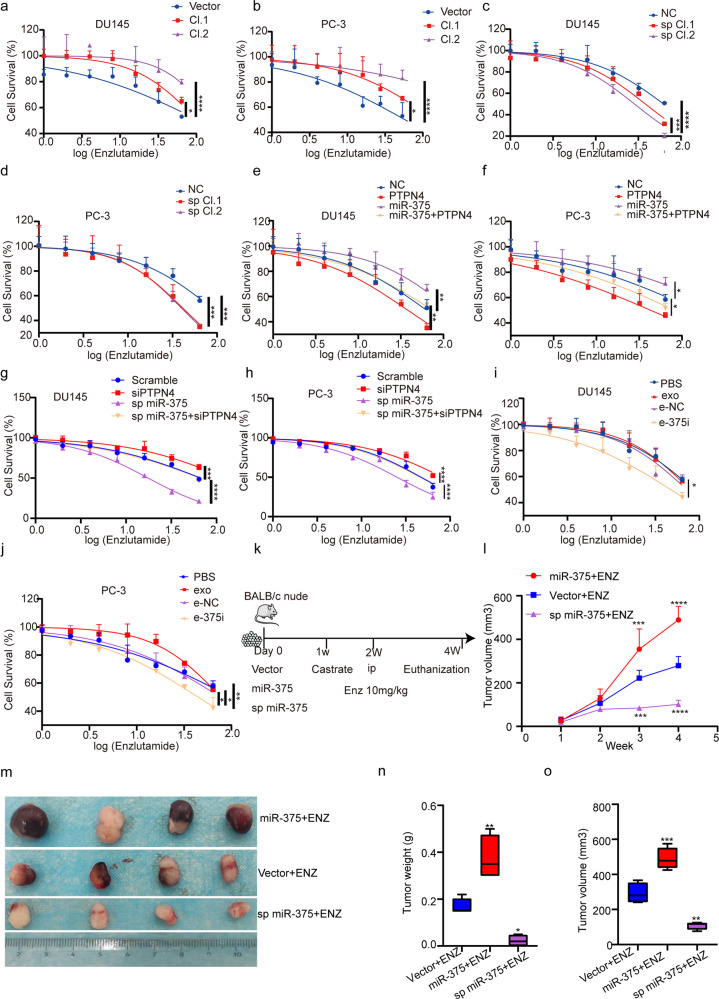
MiR-375 regulated enzalutamide resistance by targeting the PTPN4-STAT3 signaling pathway in vitro and in vivo. CCK-8 assays were conducted to test enzalutamide resistance of DU145 and PC-3 cells in response to **a**, **b** stably upregulated expression of miR-375, **c**, **d** stable knockdown with miR-375 sponge, **e**, **f** overexpression of miR-375 and/or PTPN4, **g**, **h** suppression of miR-375 and/or PTPN4, and **i**, **j** the use of miR-e-375i. **k** A flow chart showing the in vivo experimental design. **l** Tumor volume during follow-up for 4 weeks. **m** Representative images of tumors in nude mice. **n** Final tumor weight, **o** final tumor volume. ENZ: enzalutamide.

We next asked whether miR-375 targeted PTPN4/STAT3 and facilitated resistance to AR antagonists in PCa. Figure 9a–d shows that miR-375-overexpressing DU145 and PC-3 cells were more resistant to enzalutamide, while miR-375 knockdown enhanced their sensitivity. In parallel, PTPN4 overexpression in PCa cells reversed the miR-375-induced insensitivity (Fig. 9e, f) with downregulation of p-STAT3 (Fig. 4g), while PTPN4 depletion in PCa cells reversed the miR-375 inhibition-enhanced sensitivity (Fig. 9g, h), and e-375i also significantly reduced PCa cell enzalutamide resistance (Fig. 9i, j). In the DU145 xenograft nude mouse model (Fig. 9k), treatment consisting of enzalutamide and a miR-375 sponge led to the most significant inhibitory effects, as evidenced by decreased tumor sizes, tumor masses, and tumor volumes (Fig. 9l–o). The above findings collectively support the notion that miR-375 enhances AR expression through PTPN4/STAT3 signaling, manipulating AR expression changes sensitivity to androgen antagonists, even in androgen-independent PCa cells.

## Discussion

MiR-375 functions in a tissue- and organ-specific pattern in cancer. Recent studies have demonstrated that miR-375 functions as a tumor suppressor in some cancers^25–29^. In PCa, Kunz et al. found that transfection of miR-375 ASOs inhibited PCa cell growth, indicating that miR-375 is an oncomiR^30^. Interestingly, the exact function of miR-375 remains controversial even in the same PCa cell line across different studies. For example, one study indicated that ectopic expression of miR-375 promoted PC-3 apoptosis and inhibited cell viability^9^, while another study demonstrated that miR-375 enhanced PC-3 proliferation, migration, and invasion^31^_._ In this study, the gain- and loss-of-function (either by miR-375 sponge or by miR-375 antisense PMO) reciprocally revealed the important roles of miR-375 in PCa. The gain-of-function assays showed that stable upregulation of miR-375 in PCa cells promoted proliferation, migration, invasion, and enzalutamide resistance and inhibited apoptosis; on the other hand, the loss-of-function assays confirmed the results of the gain-of-function assays. Our results clearly suggested that miR-375 served as an oncomiR in prostate cancer. This argument is further supported by the following evidence: 1) bioinformatics analysis illustrating that miR-375 was enriched in prostate cancer compared with normal tissues; 2) positive association of miR-375 with tumor Gleason score; 3) the significantly higher levels of miR-375 in the PCa tissues and in the serum of PCa patients compared to benign prostate hyperplasia^32^_._

Accumulating evidence has revealed that miR-375 can impact the progression of prostate cancer by inhibiting different target genes^33,34^. For example, Selth et al. showed that miR-375 suppressed the invasion and migration of prostate cancer cells by targeting YAP1 and ZEB1 and that inhibiting the transcription of miR-375 could reverse these effects^33^. Moreover, Choi et al. demonstrated that miR-375, miR-93, and miR-106b coregulate the CIC-CRABP1 axis to facilitate the progression of prostate cancer^35^. For the first time, we identified PTPN4 as a novel direct target of miR-375. Our results demonstrated that both the protein and mRNA levels of PTPN4 were reduced in response to miR-375 overexpression but increased after miR-375 downregulation. PTPN4 is a member of the protein tyrosine phosphatase (PTP) family^36^. Liu et al. reported that miR-15b-5p possibly promoted tumorigenesis by binding to PTPN4 and activating the STAT3 signaling pathway^18^. By GSEA, we found that PTPN4 was negatively correlated with the STAT3 pathway. This result was consistent with the report where loss of PTPN4 promoted STAT3 activity and accelerated the growth of rectal cancer^20^. STAT3 is a key player in the JAK/STAT pathway^37,38^. A number of studies have indicated that the excessive activation of STAT3 can prevent tumor cells from undergoing apoptosis and facilitate cell proliferation, invasion, and migration^39,40^. For example, Hashemi et al. recapitulated that inhibiting the p68/STAT3 pathway could suppress tumor growth, colony formation and migration^41^. Chen et al. found that STAT3 contributed to GC progression and poor prognosis via lncRNA HAGLROS/mTOR^42^. Our results demonstrated that upregulation of PTPN4 was able to attenuate the promoting effect of miR-375 on p-STAT3, suggesting that the PTPN4/STAT3 signaling axis accounted for one pathway through which miR-375 regulated PCa cell proliferation, migration, invasion, and apoptosis.

ASOs synthesized with PMOs can quickly and efficiently inhibit their miRNA counterpart, holding therapeutic potential for different diseases when a specific miRNA is overexpressed^43^. However, the electric neutral property of PMOs makes it very difficult for them to be transported into target cells by conventional cationic liposomes. This dilemma is largely resolved by the newly emerged vehicle hucMSC-derived exosomes. HucMSCs are a more favorable source of exosomes than other cells because hucMSCs can continually produce a large number of exosomes^44,45^. Jia et al. noted that hucMSC-derived exosomes loaded with miR-139-5p inhibited bladder tumorigenesis in vivo^46^. We utilized hucMSC exosomes carrying miR-375-interfering PMO oligomer (e-375i) to silence miR-375 in PCa cells and found that systematic administration of e-375i could efficiently enter PCa cells, knock down miR-375, upregulate PTPN4, and downregulate p-STAT3, eventually blocking the growth of PCa in vivo and in vitro.

Aberrant AR signaling plays a major role in CRPC development, which may gradually lead to the development of enzalutamide resistance^47^. One study showed that CREB5 enhanced AR activity and promoted PCa resistance to AR antagonists and androgen deprivation treatment^48^, which indicates the possible benefits of interfering with AR in CRPC treatment. Indeed, galiellalactone can suppress enzalutamide-resistant PCa by inhibiting the STAT3/AR signaling axis^22^. In this study, PCa cells with miR-375 overexpression were more resistant to enzalutamide with increased AR expression, whereas miR-375 depletion could induce the opposite effects. These results were notably consistent with previous reports^9,49^. Furthermore, PTPN4 overexpression reversed miR-375-induced insensitivity to enzalutamide in DU145 and PC-3 cells with a reduction in p-STAT3 and AR expression. Our study recapitulated for the first time that miR-375/PTPN4/STAT3 is an alternative axis facilitating enzalutamide resistance. Interestingly, it has been reported that AR overexpression can upregulate miR-375 expression by modulating the miR-375 promoter methylation status^49^. Therefore, miR-375 is likely to enhance enzalutamide resistance through a positive regulatory loop involving the PTPN4/STAT3/AR axis. Disrupting this loop is especially attractive and promising for PCa treatment, especially for patients whose AR level is high.

It is worth noting that the AR-negative state of DU145 and PC-3 cell lines, suggested by some recent literature^50,51^, is a technical artifact rather than a theoretical reality. Technically, western blotting is applied to evaluate AR expression using cell lysates prepared from PCa lines that traditionally include LNCaP cells, where AR expression is very high. When the protein-transferred membrane is developed with an autoexposure platform, the signal of the AR band in the LNCaP lane will be so robust as to mandatorily shorten the exposure time, eventually making PC-3 and DU145 lines appear to be devoid of AR expression. Similar results were repeatedly obtained in our study as we screened the cell lines, where AR bands in PC-3 and DU145 lanes could only be distinctively detected upon over-developing conditions when LNCaP protein was also included (Fig. 8a). In addition to the AR reference, the volume of loaded protein may be another reason for the artifact during western blotting. Many researchers, including technical experts from pharmaceutical companies, load 10–15 μg of total protein to detect specific targets. In our experiment, the AR signal could not be detected when the protein was sampled at concentrations lower than 25 μg. In support of our findings, positive expression of AR in both PC-3 and DU145 cells has been identified in many other studies^52,53^. In particular, Alimirah et al., whose study was specifically performed to determine AR expression in various PCa cells, clearly demonstrated the moderate expression of AR in PC-3 and DU145 lines^24^. Collectively, substantial evidence has fully established that PC-3 and DU145 cells are AR positive.

Despite the above findings, this study also has some limitations. For example, an essential prerequisite for the universal application of e-375i in the clinic is that miR-375 universally promotes the progression of PCa, including castration-resistant and castration-sensitive PCa. Thus, we should extend our research from AR-insensitive to AR-sensitive PCa cells, as well as PCa organoids. In addition, we found in the current study that miR-375 can inhibit apoptosis and promote drug resistance in PCa. Whether the inhibited apoptotic property is the main cause of the drug-resistant phenotype of PCa remains to be fully elucidated. Identification of the miR-375-regulated gene network may contribute to addressing this issue.

**Fig. 10:**
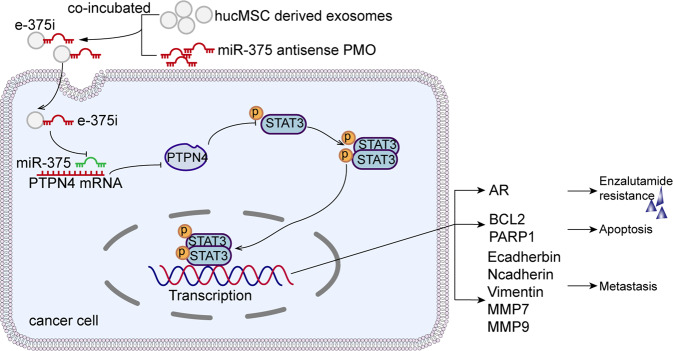
Schematic of e-375i elicited anti-PCa effects. Shown is the graphical illustration of the identified pathway of miR-375 in regulating PCa progression and enzalutamide resistance.

In summary, this is the first proof of principle study that shows that miR-375 can facilitate prostate cancer progression and enzalutamide resistance via the PTPN4/STAT3 pathway. HucMSC-derived exosomes loaded with specific antisense PMO oligomers dramatically reversed the effect of aberrantly expressed miR-375 (Fig. 10). Our findings indicate that miR-375 could serve as a novel therapeutic target for CRPC and that hucMSC-derived exosomes may serve as a safe and efficient vehicle in gene therapy.

## Supplementary information

Supplementary file

## References

[1] Sung H (2021). Global cancer statistics 2020: GLOBOCAN estimates of incidence and mortality worldwide for 36 cancers in 185 countries. CA Cancer J. Clin..

[2] Han Q (2021). Targeted inhibition of SIRT6 via engineered exosomes impairs tumorigenesis and metastasis in prostate cancer. Theranostics.

[3] Han Y (2017). Triptolide inhibits the AR signaling pathway to suppress the proliferation of enzalutamide resistant prostate cancer cells. Theranostics.

[4] Wang Y (2021). Mechanisms of enzalutamide resistance in castration-resistant prostate cancer and therapeutic strategies to overcome it. Br. J. Pharmacol..

[5] Mollaei H, Safaralizadeh R, Rostami Z (2019). MicroRNA replacement therapy in cancer. J. Cell Physiol..

[6] Huang X (2015). Exosomal miR-1290 and miR-375 as prognostic markers in castration-resistant prostate cancer. Eur. Urol..

[7] Bhagirath D (2020). MicroRNA determinants of neuroendocrine differentiation in metastatic castration-resistant prostate cancer. Oncogene.

[8] Wang Y (2016). miR-375 induces docetaxel resistance in prostate cancer by targeting SEC23A and YAP1. Mol. Cancer.

[9] Costa-Pinheiro P (2015). MicroRNA-375 plays a dual role in prostate carcinogenesis. Clin. Epigenet..

[10] Chen Z (2021). microRNA-6785-5p-loaded human umbilical cord mesenchymal stem cells-derived exosomes suppress angiogenesis and metastasis in gastric cancer via INHBA. Life Sci..

[11] Liang G (2020). Engineered exosomes for targeted co-delivery of miR-21 inhibitor and chemotherapeutics to reverse drug resistance in colon cancer. J. Nanobiotechnol..

[12] Han S (2021). Delivery of anti-miRNA-221 for colorectal carcinoma therapy using modified cord blood mesenchymal stem cells-derived exosomes. Front. Mol. Biosci..

[13] Wolfe JM (2018). Machine learning to predict cell-penetrating peptides for antisense delivery. Acs. Cent. Sci..

[14] Lobo S, Pereira C, Oliveira C, Almeida GM (2020). Skipping Exon-v6 from CD44v6-containing isoforms influences chemotherapy response and self-renewal capacity of gastric cancer cells. Cancers.

[15] Ma W (2012). Inhibition of p53 expression by peptide-conjugated phosphorodiamidate morpholino oligomers sensitizes human cancer cells to chemotherapeutic drugs. Oncogene.

[16] Gan J (2020). The influence of photodynamic therapy on the Warburg effect in esophageal cancer cells. Lasers Med. Sci..

[17] Liu C (2021). Blocking IL-17A enhances tumor response to anti-PD-1 immunotherapy in microsatellite stable colorectal cancer. J. Immunother. Cancer.

[18] Liu X, Dong Y, Song D (2020). Inhibition of microRNA-15b-5p attenuates the progression of oral squamous cell carcinoma via modulating the PTPN4/STAT3 axis. Cancer Manag. Res..

[19] Tang Z (2017). GEPIA: a web server for cancer and normal gene expression profiling and interactive analyses. Nucleic Acids Res..

[20] Zhang BD (2019). Loss of PTPN4 activates STAT3 to promote the tumor growth in rectal cancer. Cancer Sci..

[21] Gao L (2021). KIF15-mediated stabilization of AR and AR-V7 contributes to enzalutamide resistance in prostate cancer. Cancer Res..

[22] Thaper D (2018). Galiellalactone inhibits the STAT3/AR signaling axis and suppresses Enzalutamide-resistant Prostate Cancer. Sci. Rep..

[23] Sampson N (2018). Inhibition of Nox4-dependent ROS signaling attenuates prostate fibroblast activation and abrogates stromal-mediated protumorigenic interactions. Int. J. Cancer.

[24] Alimirah F, Chen J, Basrawala Z, Xin H, Choubey D (2006). DU-145 and PC-3 human prostate cancer cell lines express androgen receptor: implications for the androgen receptor functions and regulation. FEBS Lett..

[25] Wu Y, Sun X, Song B, Qiu X, Zhao J (2017). MiR-375/SLC7A11 axis regulates oral squamous cell carcinoma proliferation and invasion. Cancer Med..

[26] Jayamohan S (2019). Dysregulation of miR-375/AEG-1 axis by human papillomavirus 16/18-E6/E7 promotes cellular proliferation, migration, and invasion in cervical cancer. Front. Oncol..

[27] Xu X (2019). miR-375-3p suppresses tumorigenesis and partially reverses chemoresistance by targeting YAP1 and SP1 in colorectal cancer cells. Aging.

[28] He Z, Li W, Zheng T, Liu D, Zhao S (2020). Human umbilical cord mesenchymal stem cells-derived exosomes deliver microRNA-375 to downregulate ENAH and thus retard esophageal squamous cell carcinoma progression. J. Exp. Clin. Cancer Res..

[29] Xu X (2021). Selective exosome exclusion of miR-375 by glioma cells promotes glioma progression by activating the CTGF-EGFR pathway. J. Exp. Clin. Cancer Res..

[30] Kunz M (2020). Nanoparticle-complexed antimiRs for inhibiting tumor growth and metastasis in prostate carcinoma and melanoma. J. Nanobiotechnol..

[31] Pickl JM (2016). Ago-RIP-Seq identifies Polycomb repressive complex I member CBX7 as a major target of miR-375 in prostate cancer progression. Oncotarget.

[32] Abramovic I (2021). MiR-182-5p and miR-375-3p have higher performance than PSA in discriminating prostate cancer from benign prostate hyperplasia. Cancers.

[33] Selth LA (2017). A ZEB1-miR-375-YAP1 pathway regulates epithelial plasticity in prostate cancer. Oncogene.

[34] Pillman KA (2018). miR-200/375 control epithelial plasticity-associated alternative splicing by repressing the RNA-binding protein Quaking. Embo j..

[35] Choi N (2015). miR-93/miR-106b/miR-375-CIC-CRABP1: a novel regulatory axis in prostate cancer progression. Oncotarget.

[36] Caillet-Saguy C (2017). Regulation of the human phosphatase PTPN4 by the inter-domain linker connecting the PDZ and the phosphatase domains. Sci. Rep..

[37] Jin W (2020). Role of JAK/STAT3 signaling in the regulation of metastasis, the transition of cancer stem cells, and chemoresistance of cancer by epithelial-mesenchymal transition. Cells.

[38] Johnson DE, O’Keefe RA, Grandis JR (2018). Targeting the IL-6/JAK/STAT3 signalling axis in cancer. Nat. Rev. Clin. Oncol..

[39] Hua K (2020). Long noncoding RNA HOST2, working as a competitive endogenous RNA, promotes STAT3-mediated cell proliferation and migration via decoying of let-7b in triple-negative breast cancer. J. Exp. Clin. Cancer Res..

[40] Yang L (2019). Novel activators and small-molecule inhibitors of STAT3 in cancer. Cytokine Growth Factor Rev..

[41] Hashemi V (2020). Silencing of p68 and STAT3 synergistically diminishes cancer progression. Life Sci..

[42] Chen JF (2018). STAT3-induced lncRNA HAGLROS overexpression contributes to the malignant progression of gastric cancer cells via mTOR signal-mediated inhibition of autophagy. Mol. Cancer.

[43] Bajan S, Hutvagner G (2020). RNA-based therapeutics: from antisense oligonucleotides to miRNAs. Cells.

[44] Wang Y (2021). miR-224-5p carried by human umbilical cord mesenchymal stem cells-derived exosomes regulates autophagy in breast cancer cells via HOXA5. Front. Cell Dev. Biol..

[45] Yao Z (2021). MicroRNA engineered umbilical cord stem cell-derived exosomes direct tendon regeneration by mTOR signaling. J. Nanobiotechnol..

[46] Jia Y, Ding X, Zhou L, Zhang L, Yang X (2021). Mesenchymal stem cells-derived exosomal microRNA-139-5p restrains tumorigenesis in bladder cancer by targeting PRC1. Oncogene.

[47] Watson PA, Arora VK, Sawyers CL (2015). Emerging mechanisms of resistance to androgen receptor inhibitors in prostate cancer. Nat. Rev. Cancer.

[48] Hwang JH (2019). CREB5 promotes resistance to androgen-receptor antagonists and androgen deprivation in prostate cancer. Cell Rep..

[49] Chu M (2014). Androgen receptor is negatively correlated with the methylation-mediated transcriptional repression of miR-375 in human prostate cancer cells. Oncol. Rep..

[50] Fragni M (2019). Abiraterone acetate exerts a cytotoxic effect in human prostate cancer cell lines. Naunyn Schmiedebergs Arch. Pharm..

[51] Wang Z (2020). ELL2 is required for the growth and survival of AR-negative prostate cancer cells. Cancer Manag. Res..

[52] Giatromanolaki A (2019). CYP17A1 and androgen-receptor expression in prostate carcinoma tissues and cancer cell lines. Curr. Urol..

[53] Jacob S (2014). Androgen receptor as a regulator of ZEB2 expression and its implications in epithelial-to-mesenchymal transition in prostate cancer. Endocr. Relat. Cancer.

