# Supplementary material for: MicroRNA-375 is a therapeutic target for castration-resistant prostate cancer through the PTPN4/STAT3 axis
Source: Exp Mol Med. 2022 Aug 30;54(8):1290–305. doi: 10.1038/s12276-022-00837-6 (PMC9440249; doi:10.1038/s12276-022-00837-6)
Supplement: Supplementary file 1 — Supplementary file [file 12276_2022_837_MOESM1_ESM.docx]

Supplementary Fig.1


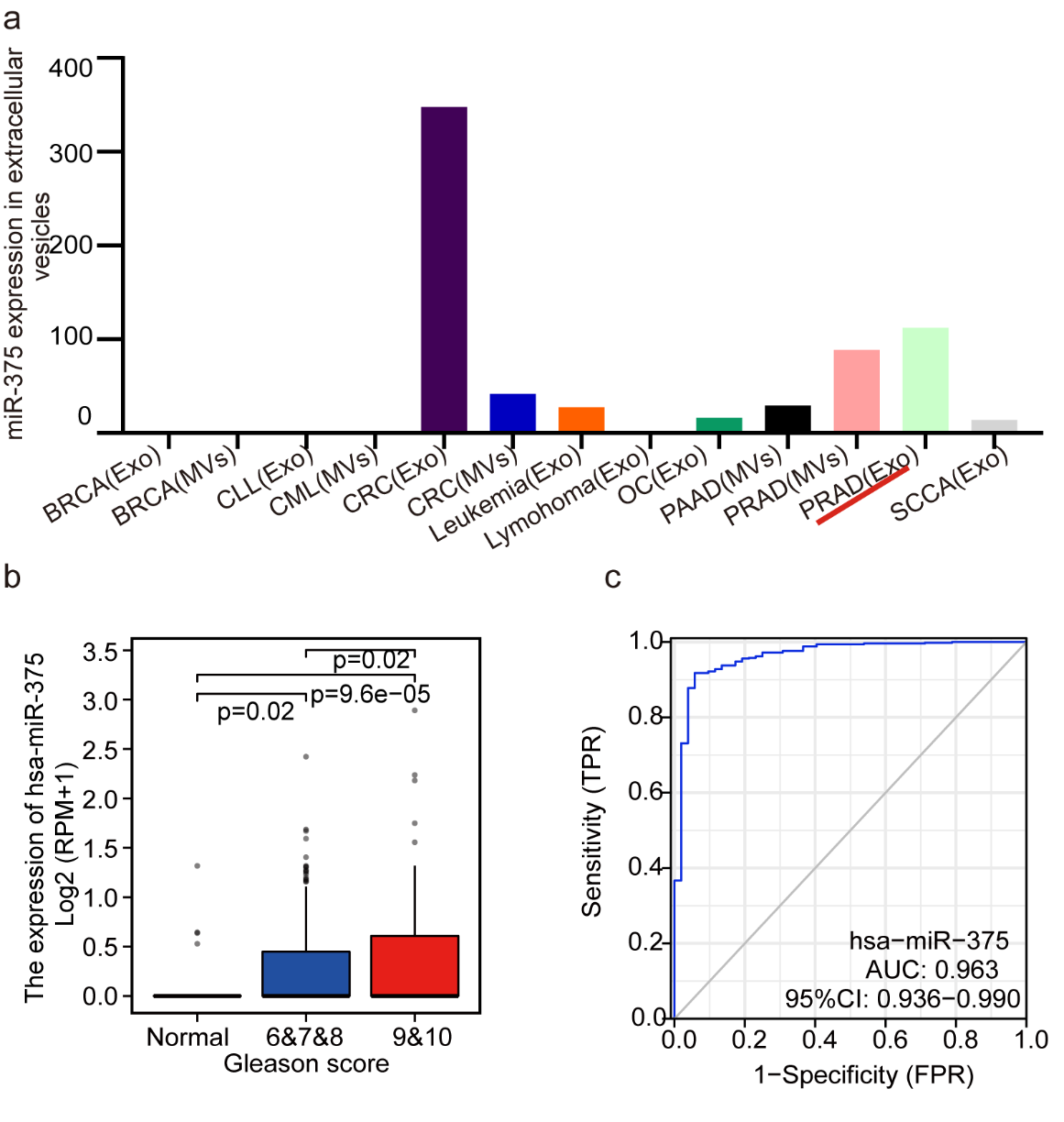


Supplementary Fig.2


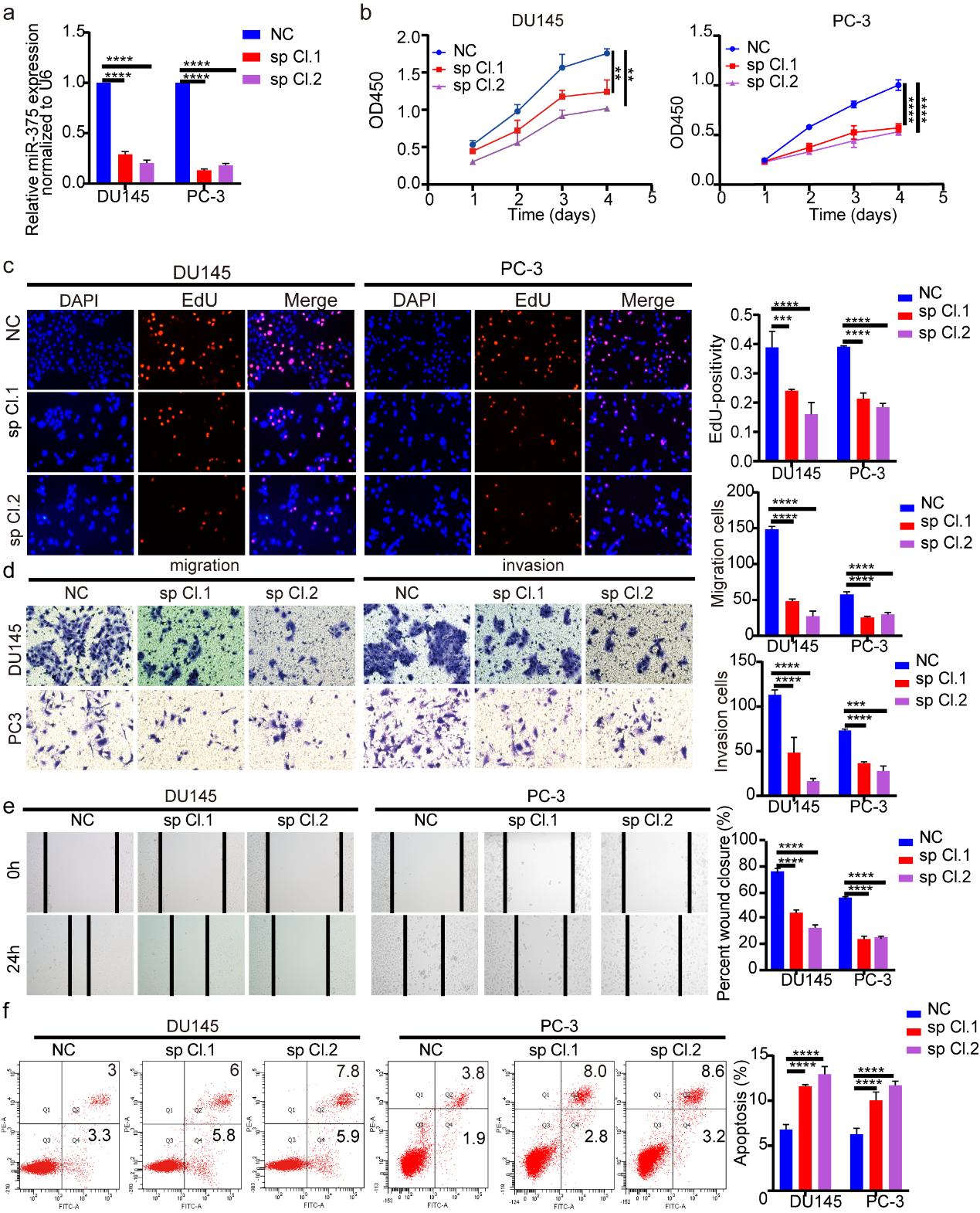


Supplementary Fig.3


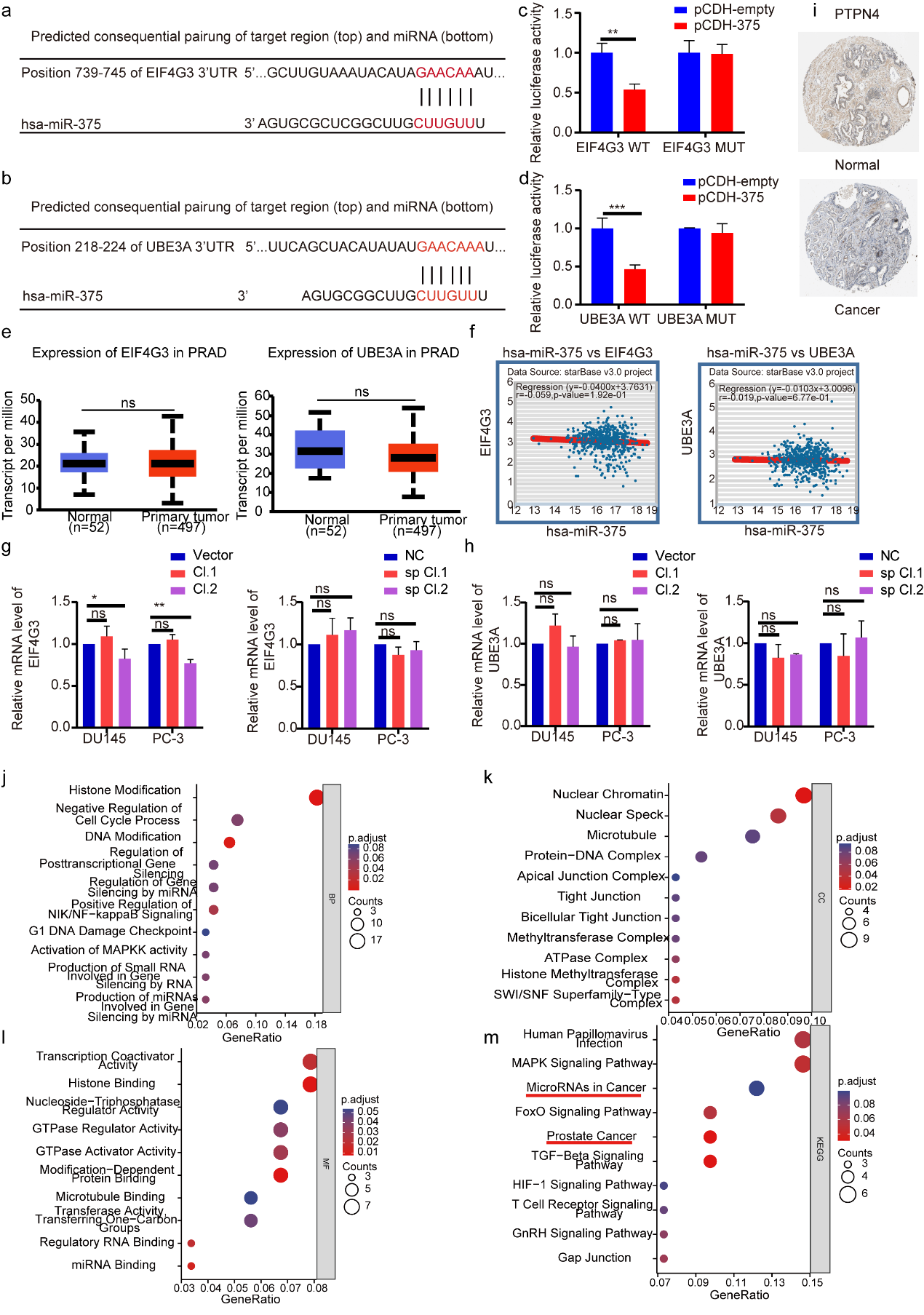


Supplementary Fig.4


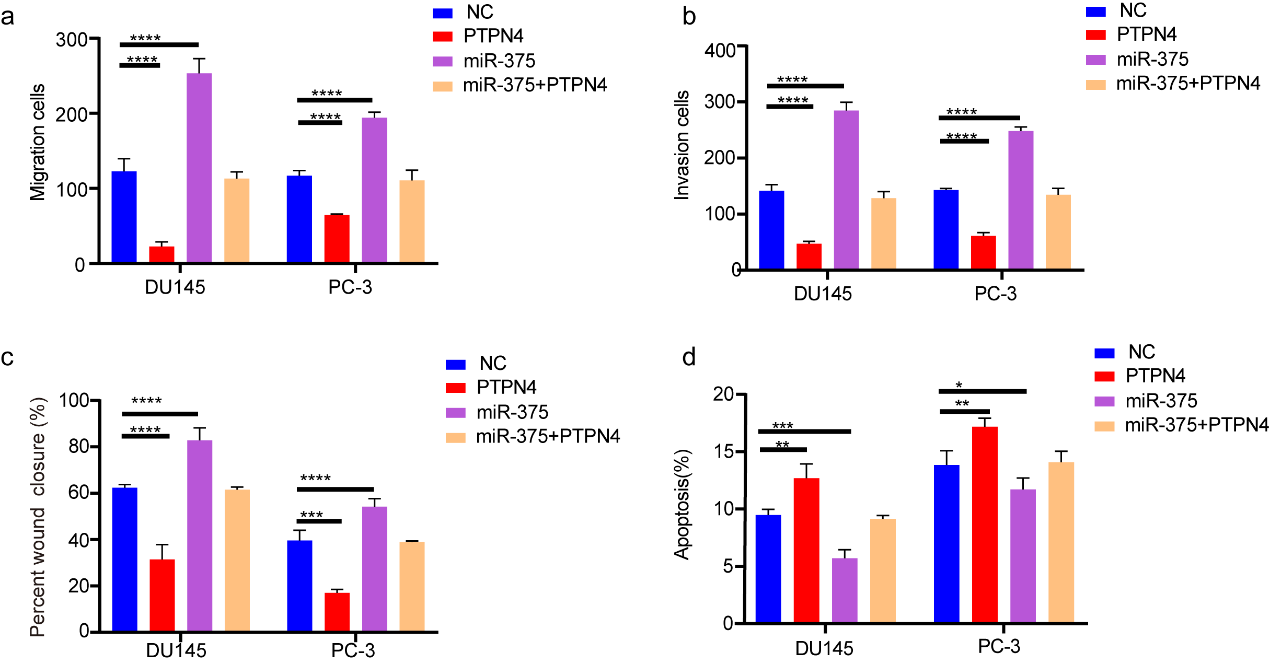


Supplementary Fig.5


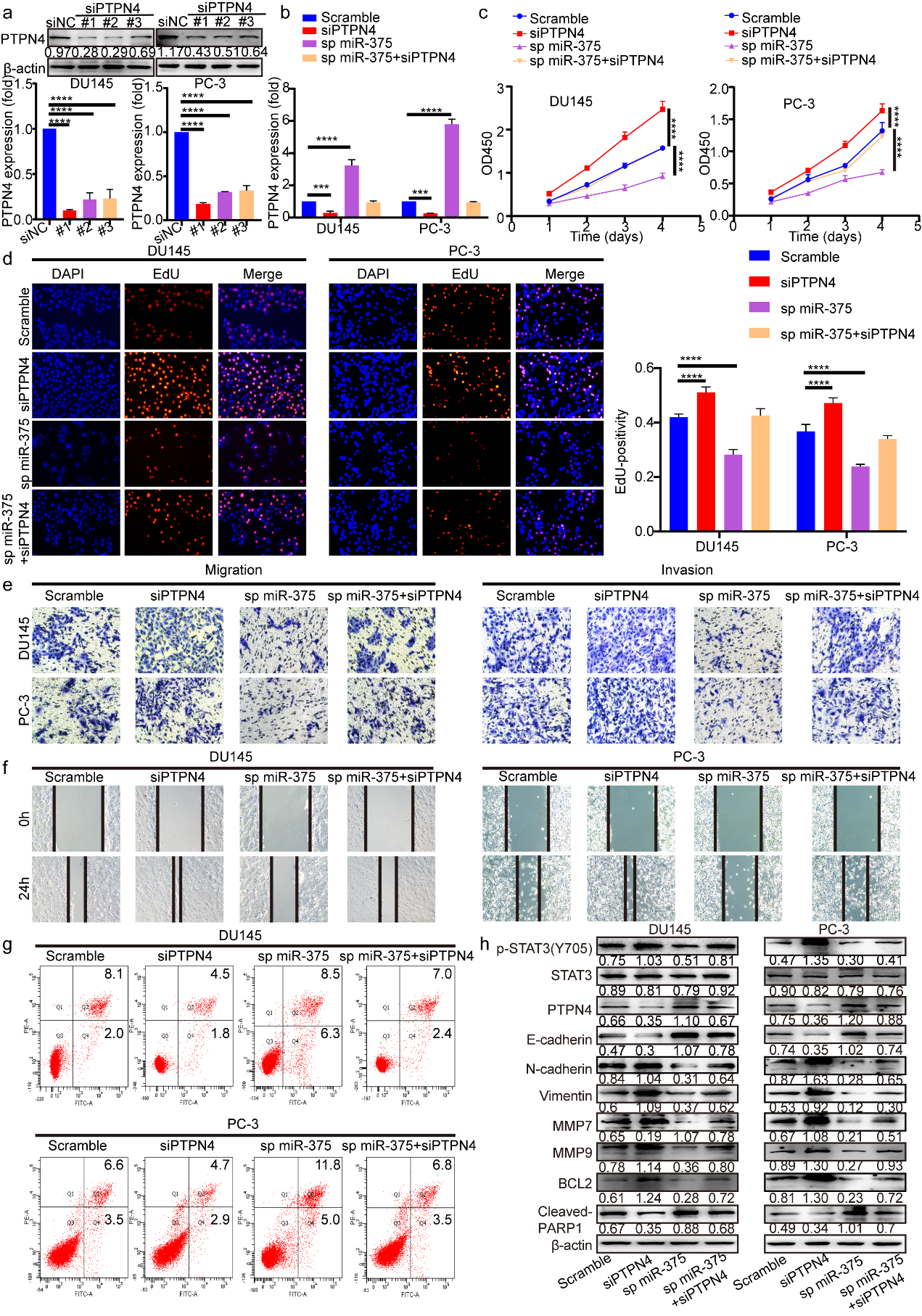


Supplementary Fig.6


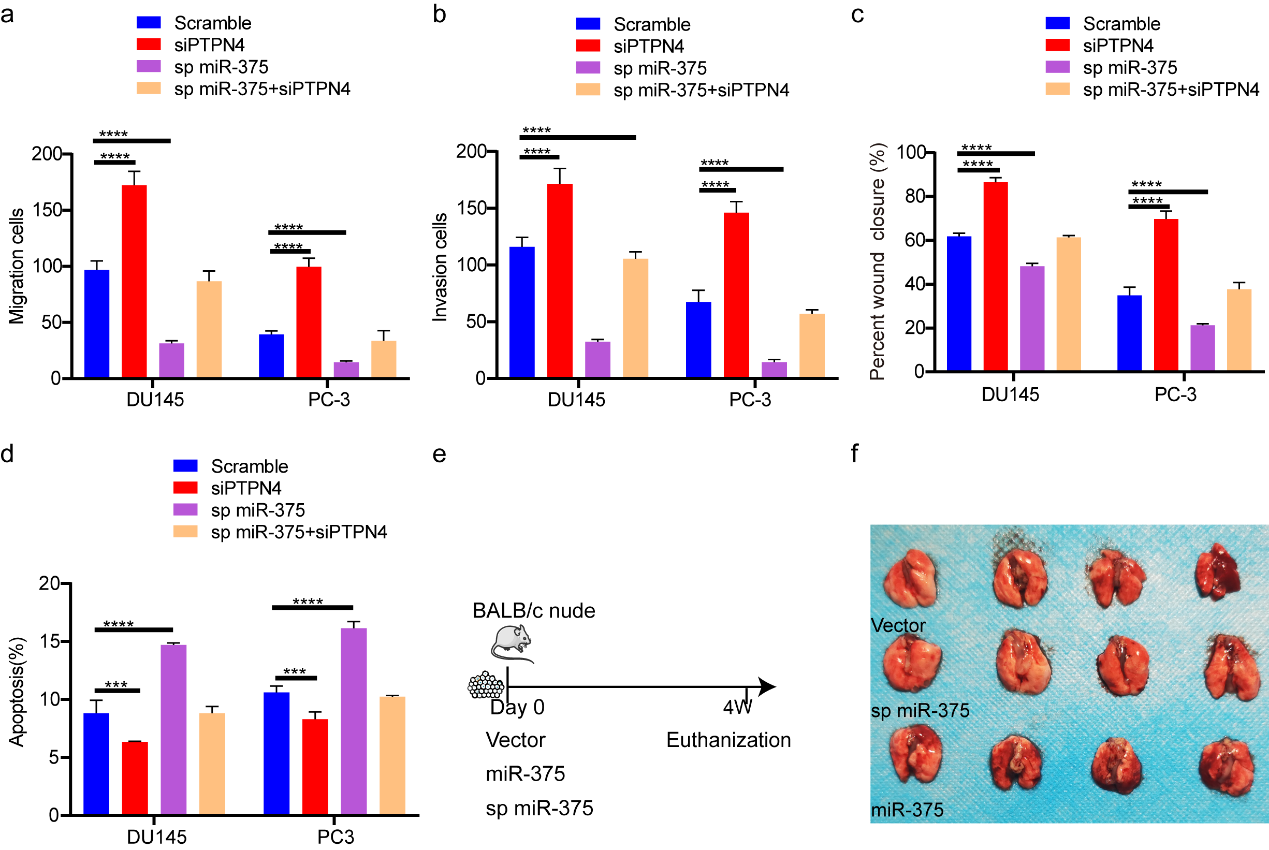


Supplementary Fig.7


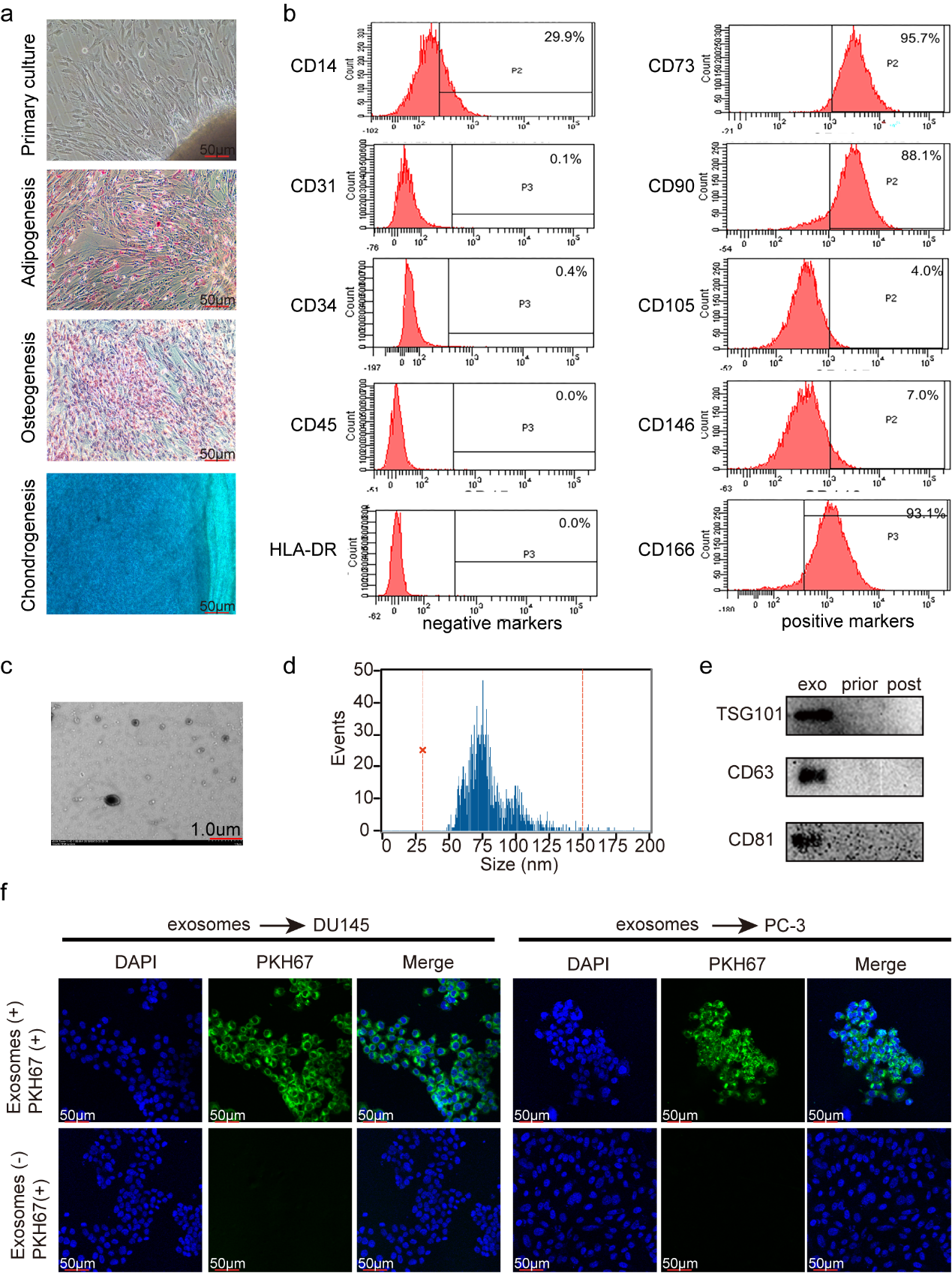


Supplementary Fig.8


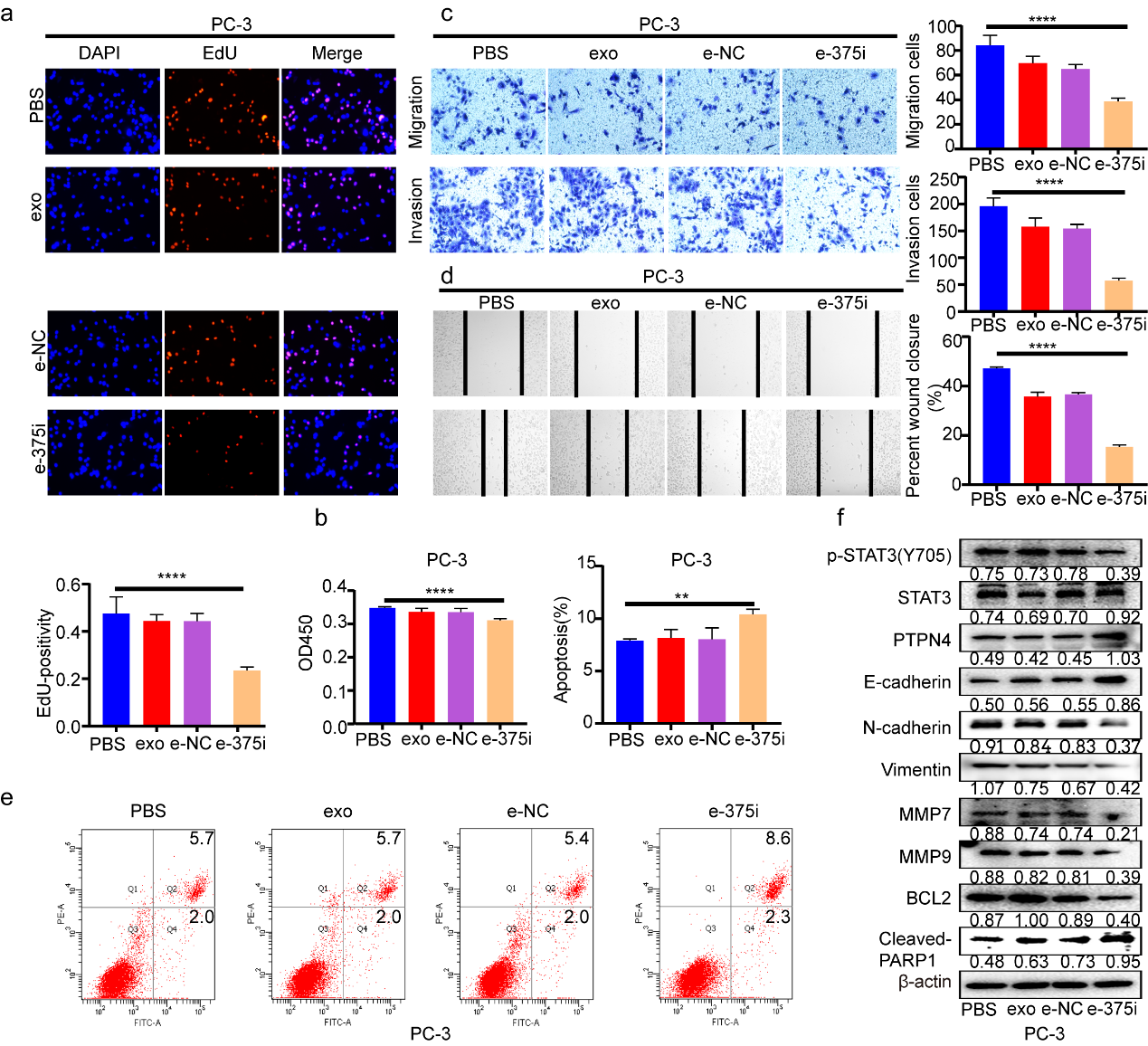


Supplementary Fig.1 MiR-375 level in exosome and its significance in PCa. (a) Expression levels of miR-375 in exosomes derived from various cancer in EvmiRNA database. (b) The correlation of miR-375 level to Gleason Score of PCa calculated using data from TCGA. (c) Receiver operating characteristic (ROC) analysis illustrating the diagnostic power of miR-375 expression for PCa using the data from TCGA database. AUC, area under the curve; Exo: exosome; MVs: microvesicles. BRCA: breast cancer; CLL: chronic lymphocytic leukemia; CML: chronic myeloid leukemias; CRC: colorectal cancer; OC: ovarian cancer; PAAD: pancreatic adenocarcinoma; PRAD: prostate cancer; SCCA: squamous cell carcinoma.

Supplementary Fig.2 Inhibiting miR-375 expression reduced PCa cell proliferation, migration, invasion and accelerated apoptosis. (a) MiR-375 knockdown in DU145 and PC-3 were verified via qRT-PCR after these cells were stably transfected with miR-375 sponge or scramble sponge vector (serve as control). CCK-8 and EdU incorporation assays were performed to assess the cell proliferation (b) and DNA synthesis rate (c) in miR-375 knocked-down DU145 and PC-3 cells. Transwell (d) and wound healing assay (e) were used to confirm the effects of miR-375 depletion on cell migration and invasion. (f) Flow cytometric analysis on the rate of cell apoptosis in DU145 and PC-3 cells with or without miR-375 knockdown. sp Cl.1: cell colony 1 transfected with pHB-U6-MCS-PGK-PURO-miR-375 sponge vector. Sp Cl.2: cell colony 2 transfected with pHB-U6-MCS-PGK-PURO-miR-375 sponge vector. NC: cell colony transfected with pHB-U6-MCS-PGK-PURO-empty vector (serve as control).

Supplementary Fig.3 Identification of the target of miR-375 and enrichment analysis of PTPN4. (a)(b) Potential binding site of miR-375 with the 3’-UTR of EIF4G3 (a) and UBE3A (b)was predicted using Targetscan database. (c)(d) Luciferase assay was conducted to validate the direct binding and modulating function of miR-375 to 3’-UTR of EIF4G3 (c) and UBE3A (d). PCDH-empty: HEK-293 cells that transfected with pCDH-CMV vector. PCDH-375: HEK-293 cells transfected with pCDH-CMV vector that was subcloned with fragment of pri-miR-375. EIF4G3 WT or UBE3A WT: HEK-293 cells that co-transfected with P-MIR-Report firefly luciferase vector that inserted with fragment of wild type 3ʹUTR of EIF4G3 or UBE3A mRNA. EIF4G3 or UBE3A MUT: HEK-293 cells that co-transfected with P-MIR-Report firefly luciferase vector subcloned with 3’-UTR of EIF4G3 or UBE3A mRNA mutated at the complementary site. (e) The expression of EIF4G3 and UBE3A in PCa tissues compared with normal prostate tissues from UALCAN database. (f) The correlation between miR-375 level and EIF4G3 and UBE3A in PCa, statistically processed by Starbase (r=-0.059, p=1.92e-1; r=-0.019, p=6.77e-1, respectively). (g) EIF4G3 and (h) UBE3A were explored in mRNA level by qRT-PCR in DU145 and PC-3 after miR-375 was overexpressed or knocked-down. (i) Represent IHC image illustrating expression of PTPN4 in prostate cancer tissues and normal tissues in HPA database. Go analysis showed the biological processes(j), cellular components (k), and molecular functions (l) that PTPN4 might be involved or possessed. (m) Signaling pathways that PTPN4 may be involved, predicted by KEGG database.

Supplementary Fig.4 Statistical analysis of results derived from the transwell assay without Matrigel(a), the transwell assay with Matrigel(b), the wound-healing assay(c), and apoptosis analysis(d) in DU145 and PC-3 cells in response to overexpression of miR-375 and/or PTPN4. NC: cells stably transfected with pSUPER-RETRO-Puro-empty vector and transiently transfected with pcDNA3.1-empty vector. PTPN4: cells stably transfected with pSUPER-RETRO-Puro-empty vector and transiently transfected with pcDNA3.1-PTPN4 recombinant vector. MiR-375: cells stably transfected with pSUPER-RETRO-Puro-miR-375 recombinant vector and transiently transfected with pcDNA3.1-empty vector. MiR-375 + PTPN4: cells stably transfected with pSUPER-RETRO-Puro-miR-375 recombinant vector and transiently transfected with pcDNA3.1-PTPN4 vector.

Supplementary Fig.5 Additional PTPN4 rescue assay by co-transfection of miR-375 sponge and PTPN4 siRNA showed anti-miR-375 effects of PTPN4 in PCa cells. (a) Inhibition of PTPN4 in DU145 and PC-3 cells by three siRNA was tested by qRT-PCR and western blot. (b) QRT-PCR was used to detect mRNA expression of PTPN4 post PTPN4 and/or miR-375 inhibition. (c) CCK-8 and (d) EDU incorporation assays were conducted to test proliferation of DU145 and PC-3 in response to suppression of miR-375 and/or PTPN4. (e) Transwell assay and (f) wound-healing assay was used to test migration and invasion of DU145 and PC-3 in response to suppression of miR-375 and/or PTPN4. (g) Apoptotic rate of DU145 and PC-3 in response to miR-375 and/or PTPN4 suppression, determined by flow cytometry. (h) Western blot was conducted to test p-STAT3，PTPN4, EMT and apoptosis related markers in DU145 and PC-3 influenced by suppression of miR-375 and/or PTPN4. Scramble: cells stably transfected with pHB-U6-MCS-PGK-PURO-NC and transiently transfected with siNC. SiPTPN4: cells stably transfected with pHB-U6-MCS-PGK-PURO-NC and transiently transfected with siPTPN4 recombinant vector. Sp miR-375: cells stably transfected with pHB-U6-MCS-PGK-PURO-miR-375 sponge and transiently transfected with siNC. Sp miR-375 + siPTPN4: cells stably transfected with pHB-U6-MCS-PGK-PURO-miR-375 sponge recombinant vector and transiently transfected with siPTPN4 vector.

Supplementary Fig.6 PTPN4 showed anti-miR-375 effects in PCa cells, continued. Statistical analysis of results derived from the transwell assay without Matrigel(a), the transwell assay with Matrigel(b), the wound-healing assay(c), and apoptosis analysis(d) in DU145 and PC-3 cells in response to suppression of miR-375 and/or PTPN4. (e)The flow chart showing in vivo experimental design. (f) Representative lung images. Scramble: cells stably transfected with pHB-U6-MCS-PGK-PURO-NC and transiently transfected with siNC. SiPTPN4: cells stably transfected with pHB-U6-MCS-PGK-PURO-NC and transiently transfected with siPTPN4 recombinant vector. Sp miR-375: cells stably transfected with pHB-U6-MCS-PGK-PURO-miR-375 sponge and transiently transfected with siNC. Sp miR-375 + siPTPN4: cells stably transfected with pHB-U6-MCS-PGK-PURO-miR-375 recombinant vector and transiently transfected with siPTPN4 vector. Vector: DU145 cells stably transfected with pSUPER-RETRO-Puro-empty vector and pHB-U6-MCS-PGK-PURO-empty vector. MiR-375: DU145 cells stably transfected with pSUPER-RETRO-Puro-miR-375 vectorand pHB-U6-MCS-PGK-PURO-empty vector. Sp miR-375: DU145 cells stably transfected with pHB-U6-MCS-PGK-PURO-miR-375 sponge vector and pSUPER-RETRO-Puro-empty vector.

Supplementary Fig.7 Identification of the hucMSC and hucMSC derived exosomes. (a)The morphology of the primarily cultured hucMSC (top), whose capabilities in adipogenesis (upper in the middle), osteogenesis (lower in the middle), and chondrogenesis (bottom) were respectively determined with Oil red O, Alizarin red S, and Alcian blue staining at the 4th passage. (b) Flow cytometry was used to detect expression of hucMSC surface markers. (c)TEM images of exosomes isolated from supernatant of hucMSC culture. (d) Nano-flow analysis on the size distribution and concentration of the hucMSC exosomes. (e) Western blot analysis of exosome specific markers using the exosomes, the supernatant prior to and post ultracentrifugation. (f) Confocal microscopy image of the internalization of PKH67 labeled exosomes in PCa cells.

Supplementary Fig.8 E-375i inhibited prostate cancer proliferation, migration, invasion while promoted apoptosis in PC-3 cells. (a) EdU incorporation and (b) CCK-8 assays were used to assess the role of miR-375 AMO loaded exosomes on proliferation in PC-3 cells. (c)Transwell and (d) wound-healing assays were applied to test the effects of miR-375 AMO on cell migration and invasion of PC-3 cells. (e) Flow cytometry was used to test the function of miR-375 AMO on apoptotic rate of of PC-3 cells. (f) The effect of miR-375 AMO on protein expression of p-STAT3, EMT and apoptosis markers of PC-3 cells. PBS: cell treated with PBS that serve as resuspendant of exosomes. NC: scramble oligonucleotides. exo: exosomes suspended in PBS. e-NC: exosomes loaded with scramble oligonucleotides. e-375i: exosomes loaded with miR-375 AMO.

Supplementary Table 1. Primers used for real-time PCR

| Gene name | Forward | Reverse |
| --- | --- | --- |
| miR-375 | 5’-TTTGTTCGTTCGGCTCGC-3’ | 5’-CAGTGCGTGTCGTGGAGT-3’ |
| U6 | 5’-CTCGCTTCGGCAGCACA-3’ | 5’-AACGCTTCACGAATTTGCGT-3’ |
| PTPN4 | 5’-TCAGAAGATGCCTGTGATTGTGT-3’ | 5’-TGTCCCGACCATTGATCAGTAC-3’ |
| GAPDH | 5’-CACCCACTCCTCCACCTTTGA-3’ | 5’-ACCACCCTGTTGCTGTAGCCA-3’ |
| AR  UBE3A  EIF4G3 | 5’-CAGCCTATTGCGAGAGAGCTG-3’  5’-ACGACATTGAAGCTAGCCGAAT-3’  5’-TACCCAAGTCAGCCGGTGTAT-3’ | 5’-GAAAGGATCTTGGGCACTTGC-3’  5’-TGGACAGGAAGCACAAAACTCA-3’  5’-TGGTTTGGATCCCGAATTCTT-3’ |

Supplementary Table 2. Protein antibodies used for western blot, IHC and flow cytomety.

| Antibody | Manufacturer | Product code |
| --- | --- | --- |
| E-cadherin | Proteintech | 20874-1-AP |
| N-cadherin | Proteintech | 66219-1-Ig |
| Vimentin | Proteintech | 60330-1-Ig |
| MMP7 | Proteintech | 10374-2-AP |
| MMP9 | Proteintech | 10375-2-AP |
| PTPN4 | Proteintech | 11131-1-AP |
| AR | Proteintech | 22089-1-AP |
| PARP1 | Proteintech | 13371-1-AP |
| BCL2 | Proteintech | 12789-1-AP |
| Ki67 | Proteintech | 27309-1-AP |
| β-actin | Proteintech | 20536-1-AP |
| Goat anti-Rabbit IgG | Proteintech | B900210 |
| HRP-conjugated Affinipure Goat Anti-Mouse IgG(H+L) | Proteintech | SA00001-1 |
| P-STAT3 | Cell Signaling Technology | #9145 |
| STAT3 | Cell Signaling Technology | #4904 |
| APC Mouse Anti-Human HLA-DR | BD Pharmingen ^TM^ | 559866 |
| PE anti-human CD73 | BioLegend | 344004 |
| PE anti-human CD166 | BioLegend | 343904 |
| PE anti-CD105(Endoglin) | BioLegend | 800503 |
| PE anti-human CD146 | BioLegend | 361006 |
| PE anti-human CD90(Thy1) | BioLegend | 328110 |
| FTTC anti-human CD14 | BioLegend | 367115 |
| APC anti-human CD34 | BioLegend | 343510 |
| APC anti-human CD31 | BioLegend | 303116 |
| APC anti-human CD45 | BioLegend | 368511 |

Supplementary Table 3. Primers for sub-cloning pri-miR-375 and the 3’-UTR fragments of 3 putative targets of miR-375, predicted by 5 different databases

| Gene | Accession | Type | Primers and target sequences | |
| --- | --- | --- | --- | --- |
| miR-375 | MI0000783 | Pri-miRNA | F_BamH1: 5’-cgggatcccgGACGTGTCAGCCGCAGATG-3’ | R_EcoR1: 5’-cggaattcTACGACGCAGAATGGAGCC-3’ |
|  |  | Amplified Target | GACGTGTCAGCCGCAGATGCGTTCAGGTGAGGGCGGAGGCTAGCGGGGCGCTGTGCAGCACTGAGCTCGCGGAAGACCAGGACCAGGAGATCACCGAGGGCGACCGCCAGGCCCCGGGCCCTCCGCTCCCG***CCCCGCGACGAGCCCCTCGCACAAACCGGACCTGAGCGTTTTGTTCGTTCGGCTCGCGTGAGGC***AGGGGCGGCCTCTCAGCACCAGCCCGGGGGCCGGCCTGATCGCCACGCAGGCACCTGCCGCCGCCACCGCCACCGCCATCTCAACCGTACGGGTGGGAGAGGCTGTGCGCCGCTCCAGGGGAGATCCGGCTCCCATCCGGCCCCACCCGCCCTGCCTTGCCCTGCCCGCAGCTTCTGGGCTGCCAGGCTCCATTCTGCGTCGTA | |
| PTPN4 | NM_002830.4 | WT | F_SpeI: 5’-attactagtGCTTTCCCTTATGTTCACTGTG -3’ | R_Hind: 5’-tataagcttAGCTGACTCCTGATGTTTACTTCT-3’ |
|  |  | MUT | F: 5’-TTCATGCTTTGCTCCCTTGTTTTAGTAAATAACTG-3’ | R: 5’-AAACAAGGGAGCAAAGCATGAAACAGTCTTCA-3’ |
|  |  | Amplified Target | GCTTTCCCTTATGTTCACTGTGCCATAATGCTGCTCGCAGGAAATGGCATTTTACAAAAAAAAAATGAAGAACTCAAAAAAACTTTGAAAACTTCAGCACTGTTGCACTTTATGTTTTAAAAAATGTCACTCTTTCAAAATCTATAACTCATGTATTTGAAGACTGTTTCATGCTTTGCTC***CGAACAAA***TAGTAAATAACTGAGTATGTTCAGGGTAATTTATGAAATTTTGTGGTGGTGCCATGCAATCCCCTTTTGGTAGAATTGCCACAAACAAGGCTCAAAATTCTCATCATCTCTGTTATACACCTGTATCATGAAAGCAAAAAGAAGTAAACATCAGGAGTCAGCT | |
| UBE3A | NM_130838.4 | WT | F_ SpeI: 5’-attactagtGTCTGTGCCTCCCTTCTTTATT-3’ | R_ Hind: 5’-atcaagcttTTCATTCATTTCCAGGTCAGC-3’ |
|  |  | MUT | F: 5’-TTCAGCTACATATATCTTGTTTTCCTTTATTATT-3’ | R: 5’-AAACAAGATATATGTAGCTGAAATCTGCTGTT-3’ |
|  |  | Amplified Target | GTCTGTGCCTCCCTTCTTTATTGGGGACATGTGGGCTGGAACAGCAGATTTCAGCTACATATAT***GAACAAA***TCCTTTATTATTATTATAATTATTTTTTTGCGTGAAAGTGTTACATATTCTTTCACTTGTATGTACAGAGAGGTTTTTCTGAATATTTATTTTAAGGGTTAAATCACTTTTGCTTGTGTTTATTACTGCTTGAGGTTGAGCCTTTTGAGTATTTAAAAAATATATACCAACAGAACTACTCTCCCAAGGAAAATATTGCCACCATTTGTAGACCACGTAACCTTCAAGTATGTGCTACTTTTTTGTCCCTGTATCTAACTCAAATCAGGAACTGTATTTTTTTTAATGATTTGCTTTTGAAACTTGAAGTCTTGAAAACAGTGTGATGCAATTACTGCTGTTCTAGCCCCCAAAGAGTTTTCTGTGCAAAATCTTGAGAATCAATCAATAAAGAAAGATGGAAGGAAGGGAGAAATTGGAATGTTTTAACTGCAGCCCTCAGAACTTTAGTAACAGCACAACAAATTAAAAACAAAAACAACTCATGCCACAGTATGTCGTCTTCATGTGTCTTGCAATGAACTGTTTCAGTAGCCAATCCTCTTTCTTAGTATATGAAAGGACAGGGATTTTTGTTCTTGTTGTTCTCGTTGTTGTTTTAAGTTTACTGGGGAAAGTGCATTTGGCCAAATGAAATGGTAGTCAAGCCTATTGCAACAAAGTTAGGAAGTTTGTTGTTTGTTTATTATAAACAAAAAGCATGTGAAAGTGCACTTAAGATAGAGTTTTTATTAATTACTTACTTATTACCTAGATTTTAAATAGACAATCCAAAGTCTCCCCTTCGTGTTGCCATCATCTTGTTGAATCAGCCATTTTATCGAGGCACGTGATCAGTGTTGCAACATAATGAAAAAGATGGCTACTGTGCCTTGTGTTACTTAATCATACAGTAAGCTGACCTGGAAATGAATGAA | |
| EIF4G3 | NM_001198801.2 | WT | F_SpeI: 5’-aatactagtAGTTTCACGTCTTCGCCAATC -3’ | R_Hind: 5’-attaagcttCACACTCTCTCACTCTCTCTCGTTT -3’ |
|  |  | MUT_1 | F: 5’-GCTTGTAAATACATACTTGTTTTATTTAAAAAAA-3’ | R: 5’-AAACAAGTATGTATTTACAAGCCTTAAAGTTG-3’ |
|  |  | MUT_2 | F: 5’-CTTTGAAAATATAAACTTGTTTATAAAGACAAA-3’ | R: 5’-AAACAAGTTTATATTTTCAAAGTGAAAAGAAA-3’ |
|  |  | Amplified Target | AGTTTCACGTCTTCGCCAATCACAGTGCAGCAAGGCCAATTCTCGCAGAAACCCCCACGTGTGCACGAGTGGGAGAGGGGAAAGAGAAAAAAAGGTGATCATGGAGGAAAAAGGTACTGGATAAAAGTAAACTTCAAACCTTAGGGCGGGAGCACTAAAACCAAAATACATGTATTATTTATAGAAAATATTTTCTGTTTTAATCTTTTCTTTTTAAACAAGGACTCATACTTAAAAAAATGTTTAGCAAAAAAAAAAAAAGTTGAGAACTTTTAATTTATTTTAAGGACTGCAAATGCCAGTGTAATTTTTTAATTTGCAGTTTCTGTAAACAACTTGTATAATAGAAAAGCAGAGAAATAAATTTCCCTCCCCTTCAAGATGCACCTCATGTTTGTTTTAAGGTATAGCATTTAGTCCAGATTTGAGAAAGTTTGGGGTGAACAAGGTAAGAAAGATTTTTTTTTTTTTGGCATCAAATCTTTCTGCCTGCCTCTCAGCTTGCTTCAGAAAATTTAAAAAATCACAATAGTAATCAAAACATACATAACATTGAAACAGAAGGAAATGCTGTGGACCACAGAACTCCAAGAATTGTTTAAAAAAAAAAAAGTGCTACCCTGAGAAAAGTACTCTTAATACTCTTGAAATCTTTAGAGCAACTTTAAGGCTTGTAAATACATA***GAACAAA***TATTTAAAAAAACAAAAAGAAATTGACTCAGTACTATTTCTTTTCACTTTGAAAATATAAA***GAACAAA***ATAAAGACAAACATTGCAAGTTTAAAAGAAAGTAAAGTGACTTCTCCTTTGGACAGCTGCTGCATGTGTGCCCATTCCTGGGGGTGCTGTCTGGCTATTTATTGTCTAATTCAAATCACTCCTGAGGGGAGAGAGATAAAACGAGAGAGAGTGAGAGAGTGTG | |
| Note: Characters in lower case indicate the restriction sites and protective bases; underscored characters indicate the mutant sequences of the putative core binding sites; characters in bold and italic are miR-375 stem-loop or putative miR-375 binding sites in 3’-UTR of each gene. The MUT_1 and MUT_2 primer sets are corresponding to the first and second putative binding site in the 3’-UTR of EIF4G3. Annealing temperature was 56℃ for wild type PTPN4 and UBE3A, and 58℃ for pri-miR-375 and EIF4G3. WT: wild type; MUT: mutant. | | | | |

Supplementary Table 4. Cloning primers and short interfering RNA sequences for PTPN4 (Accession No. NM_002830)

| Name | Category | Sequence |
| --- | --- | --- |
| PTPN4 | Forward | 5’-TGTGCTGGATATCTGCAGAATTCACCATGACCTCACGTTTCCG-3’ |
| PTPN4 | Reverse | 5’-AGCTTGGTACCGAGCTCGGATCCTTATTTATTTGTTGATGTTGTTAAGGG-3’ |
| hs-PTPN4-si1 | sense | 5’-GGAAUCAUCACCAUCACAATT-3’ |
| hs-PTPN4-si1 | antisense | 5’-UUGUGAUGGUGAUGAUUCCTT-3’ |
| hs-PTPN4-si2 | sense | 5’-GCUAGUUGUGAGAGACAUUTT-3’ |
| hs-PTPN4-si2 | antisense | 5’-AAUGUCUCUCACAACUAGCTT-3’ |
| hs-PTPN4-si3 | sense | 5’-GCGAGCCAUGAUGAUCCAATT-3’ |
| hs-PTPN4-si3 | antisense | 5’-UUGGAUCAUCAUGGCUCGCTT-3’ |
| NC | sense | 5’-UUCUCCGAACGUGUCACGUTT-3’ |
| NC | antisense | 5’-ACGUGACACGUUCGGAGAATT-3’ |

Supplementary Table 5. Putative target genes of miR-375 predicted by miRWalk, Targetscan, Starbasebase, miRsystem and RNA22V2

| Cumulative weighted context++ score | Target genes |
| --- | --- |
| ~ -0.1 | ORMDL2; PDE5A; KLF12; ARHGEF12; SOX6; SLC7A11; DLG3; TEAD1; NOTCH2; CDK5R1; **PTPN4**; TRPS1; TSC1; SOX12; ATXN1; BMPR2; RTF1; ZFHX4; PDPK1; EHMT1; AHR; AEBP2; ABI2; AKAP1. |
| -0.1~ -0.2 | SP1; SERBP1; INSM1; HIVEP2; EBF3; CSNK2A1; ACSL3; YWHAZ; SPAG9; TCF12; UST; QKI; LSM12; WWC2; **UBE3A**; KLF4; ZBTB20; TSC22D2; PDE4D; ELAVL2; PAX6. |
| < -0.2 | LRP5; YBX1; ISL2; **EIF4G3**; GREM2. |

Note: Genes in bold were subjected to luciferase reporter assay.

Supplementary Table 6. Genes similar to PTPN4 in GEPIA.

| Gene | Similar gene |
| --- | --- |
| PTPN4 | CREB1,ZBED6, ASH1L,MAP3K2,MYO9A,CTDSPL2,ATRX,ASXL2,CLASP1 SP1,CEP350,RSF1,RFX7,MFAP3,KLHL11,SMC1A,AGO1,MGA,TRIM44,CREBBP,UBE4A,RIF1,SMG1,PBRM1,BAZ2B,BRWD3,PIK3C2A,CNOT6L,ZNF805,SRCAP,ZNF490,DDX18,NF1,KIF2A,MAPK1,KMT2A,ARID4A,NFAT5,TRIP11MKL2,UBR2,PUM1,SBNO1,DPP8,BAZ2A,WDFY3,APPBP2,ZNF426,DDX46,ZNF148,APC,RSBN1,SPAG9,PTAR1,ARFGEF2,RNF169,RP11298J20.4,IREB2,USP9X,ATG2B,PUM2,WAC,ZMYM4,FRS2,KMT2E,EP300,ZXDA,KDM5A,MTR,TBC1D5,RASA1,SOS1,MED13L,ZNF562,WIPF2,AFF4,MED1,RICTOR,EHF,KLHL28,DLG1,UBR1,MID2,ZSCAN29,TET2,ZNF507,PRDM2,NSD1,UBXN7,NUP153,PHC3,BCLAF1,RP11159G9.5,TMOD3,LUZP1,TRPM7,GPATCH8,RP1150E11.2,ARID1B,RAPGEF2 |
